# An *in vitro* medium for modeling gut dysbiosis associated with cystic fibrosis

**DOI:** 10.1128/jb.00286-23

**Published:** 2024-01-03

**Authors:** Kaitlyn E. Barrack, Thomas H. Hampton, Rebecca A. Valls, Sarvesh V. Surve, Timothy B. Gardner, Julie L. Sanville, Juliette L. Madan, George A. O’Toole

**Affiliations:** 1 Department of Microbiology and Immunology, Geisel School of Medicine at Dartmouth, Hanover, New Hampshire, USA; 2 Section of Gastroenterology and Hepatology, Dartmouth Hitchcock Medical Center, Lebanon, New Hampshire, USA; 3 Division of Pediatric Gastroenterology, Department of Pediatrics, Dartmouth Hitchcock Medical Center, Lebanon, New Hampshire, USA; 4 Departments of Psychiatry and Pediatrics, Dartmouth Hitchcock Medical Center, Lebanon, New Hampshire, USA; 5 Geisel School of Medicine at Dartmouth, Hanover, New Hampshire, USA; University of Chicago, Chicago, Illinois, USA

**Keywords:** cystic fibrosis, medium, dysbiosis, stool, colonoscopy

## Abstract

The gut physiology of pediatric and adult persons with cystic fibrosis (pwCF) is altered relative to healthy persons. The CF gut is characterized, in part, as having excess mucus, increased fat content, acidic pH, increased inflammation, increased antibiotic perturbation, and the potential for increased oxygen availability. These physiological differences shift nutritional availability and the local environment for intestinal microbes, thus likely driving significant changes in microbial metabolism, colonization, and competition with other microbes. The impact of any specific change in this physiological landscape is difficult to parse using human or animal studies. Thus, we have developed a novel culture medium representative of the CF gut environment, inclusive of all the aforementioned features. This medium, called CF-MiPro, maintains CF gut microbiome communities, while significantly shifting nonCF gut microbiome communities toward a CF-like microbial profile, characterized by low Bacteroidetes and high Proteobacteria abundance. This medium is able to maintain this culture composition for up to 5 days of passage. Additionally, microbial communities passaged in CF-MiPro produce significantly less immunomodulatory short-chain fatty acids (SCFA), including propionate and butyrate, than communities passaged in MiPro, a culture medium representative of healthy gut physiology, confirming not only a shift in microbial composition but also altered community function. Our results support the potential for this *in vitro* culture medium as a new tool for the study of CF gut dysbiosis.

**IMPORTANCE:**

Cystic fibrosis is an autosomal recessive disease that disrupts ion transport at mucosal surfaces, leading to mucus accumulation and altered physiology of both the lungs and the intestines, among other organs, with the resulting altered environment contributing to an imbalance of microbial communities. Culture media representative of the CF airway have been developed and validated; however, no such medium exists for modeling the CF intestine. Here, we develop and validate a first-generation culture medium inclusive of features that are altered in the CF colon. Our findings suggest this novel medium, called CF-MiPro, as a maintenance medium for CF gut microbiome samples and a flexible tool for studying key drivers of CF-associated gut dysbiosis.

## INTRODUCTION

Cystic fibrosis (CF) is a hereditary condition caused by mutations in the gene encoding the cystic fibrosis transmembrane conductance regulator (CFTR) protein. Loss of CFTR function leads to disrupted chloride and bicarbonate secretion and an imbalance in hydration of mucosal surfaces (1, 2). Clinical consequences of CF include, but are not limited to, accumulation of thick mucus at epithelial surfaces, impaired mucociliary clearance due to changes in fluid and ion fluxes, exocrine dysfunction, obstruction of the airway and intestine, inflammation, and increased susceptibility to infection (2) resulting in significant multisystem morbidity and premature mortality.

While much attention has been given to CF airway disease, there is substantial evidence indicating that the intestine is also affected by this disease, as CFTR activity is essential for proper gut function (3, 4). The changes in CF intestinal physiology result in malnutrition, malabsorption of fats and vitamins, increased acidity, and poor linear growth (3, 5, 6). The dehydrated lumen of the CF gut accumulates mucus, which can significantly slow gastrointestinal motility (7), increasing the risk of delayed meconium passage and meconium ileus in newborns (3, 5). Increased mucus has also been associated with overgrowth of select organisms (1, 8) which can lead to severe gastrointestinal (GI) symptoms and weight loss. Furthermore, the CF gut is characterized by an increase in inflammation, quantified through inflammatory measures, including calprotectin (1, 3, 5, 9 – 13), and intestinal morphological abnormalities (3, 10, 13). Inflammation further exacerbates the release of reactive oxygen species (ROS) and hemoglobin-carrying oxygen, resulting in oxidative stress and elevated oxygen levels, which, in turn, contribute to ongoing inflammation within the intestine (14) with consequences for systemic inflammation.

The altered CF intestinal milieu fosters a distinct microbiota. Starting in early life, children with CF (cwCF) have significantly reduced alpha diversity based on stool 16S rRNA amplicon library (5, 14, 15) and metagenomic sequencing (5, 16). The relative age of the fecal microbiota is delayed in cwCF compared to healthy controls (5), and microbes associated with immune training are decreased in CF over the first year of life (15). Considering that the development of the gut microbiota occurs over the first three years of life, and the interaction of the microbiota with the host is essential for immune training within this timeframe, dysbiosis in this critical window has the potential for increased susceptibility to infection, inflammation, allergy, and metabolic disorders (17). Published studies indicate that this dysbiosis persists into later childhood, adolescence, and adulthood for individuals with CF (5, 14, 15, 18, 19). Consequently, this enduring dysbiosis can have adverse effects on persons with CF (pwCF) throughout their lifetime. Notable taxonomic differences observed in cwCF include a decrease in certain immune-modulating bacterial genera of the phyla Bacteroidetes (*Bacteroides*), Firmicutes (*Faecalibacterium*), Actinobacteria (*Bifidobacterium*), and Verrucomicrobia (*Akkermansia*), all positively associated with gut health (5, 14, 15, 20 – 22). There is a consistent increase in Proteobacteria (*Escherichia coli/Shigella* spp.) and select Firmicutes (*Veillonella, Clostridium*) for pwCF (5, 11, 20 – 22). These signatures of microbial dysbiosis are also observed in other intestinal conditions, such as inflammatory bowel diseases (23, 24) and in individuals chronically exposed to inorganic arsenic (25 – 27). Thus, investigation of CF-associated microbial dysbiosis likely has application to other diseases with a similar inflammatory intestinal environment.

A published study has shown that a CFTR mutation is sufficient to alter the GI microbiome in a germ-free CF mouse model (28), suggesting that CF gut physiology is causative of microbial dysbiosis, rather than correlative. A few additional studies have investigated gut microbial dysbiosis in CF animal models (28 – 32); however, it is difficult to identify the role for CF-relevant physiological features, individually or in combination, on microbial dysbiosis in an animal model due to the significant involvement of the host immune system and the likelihood that multiple factors are impacting the microbial communities. Identifying which CF-relevant physiological feature(s) drive the observed dysbiosis associated with this disease is a critical first step in developing strategies to restore intestinal homeostasis.

In this study, we sought to develop and validate a first-generation culture medium that resembles key features of the CF colon. Development and validation of a culture medium representative of the CF colon will allow for *in vitro* experimentation under more physiologically relevant conditions. Based on a thorough literature review and consultation with physicians, we modified an existing gut microbiome medium (33) to reflect CF-relevant features. We aimed to generate a first-generation CF colon medium which incorporates sources of bile, mucin, fat, oxidative stress, antibiotics, and alternative nutrient sources reported to be increased in states of intestinal inflammation (e.g., nitrate, sulfate, formate) (13, 34 – 36). We call this new medium CF-MiPro. We hypothesized that CF-MiPro, compared to MiPro alone, would (i) maintain microbial composition of CF gut microbiome samples, including maintenance of key taxa consistently associated with dysbiosis in the CF intestine, (ii) minimally shift total microbial abundance of CF samples, as measured by CFU/mL, and (iii) maintain short-chain fatty acid (SCFA) concentrations as observed in raw CF samples. Furthermore, we hypothesized that this medium may be utilized to interrogate the roles of CF-relevant physiological features in the context of nonCF-associated gut dysbiosis. Using this medium, we show that CF gut microbiota structure is largely maintained at the phylum and genus levels, relative to MiPro, and SCFA profiles are less affected by the medium than nonCF SCFA profiles. Conversely, we can use CF-MiPro to drive CF-relevant shifts in nonCF clinical samples in terms of both composition and function. This novel medium offers a flexible and inexpensive platform to study CF gut dysbiosis in the laboratory.

## RESULTS

### Formulation of CF-MiPro

To develop an *in vitro* medium representative of the nutritional conditions of the CF colon, we first reviewed the literature to determine differential physiological features described for the CF GI tract, typically via the analysis of stool, and their relevant concentration ranges. Based on these observed ranges, we categorized a “low” and “median” concentration for each feature. In early experiments including all features at the “high” concentration resulted in significant reduction in the population of many microbes, so we did not pursue experiments at the “high” concentrations.

We modified the concentrations of these features in the recipe for MiPro, an *in vitro* medium designed to mimic the conditions of the healthy colon (33). We call these CF-like media “low-CF-MiPro” and “median-CF-MiPro.” The features and respective concentrations in each medium (low, median) are as follows (see also Table 1): sulfate (0.5, 1 mM), a precursor of H_2_S, which is increased in cases of gut inflammation (34, 35); nitrate (0.5, 1 mM), a by-product of the host inflammatory response (37); formate (0.5, 1 mM), a microbially derived product increased in the inflamed gut (36); glycerol (0.5, 1%), a marker of increased fat (38, 39); Bactrim (1, 10 µM), an antibiotic commonly prescribed to cwCF in our local cohort at Dartmouth and more broadly (40); H_2_O_2_ (1, 10 µM), a measure of inflammation-derived oxidative stress (41, 42); pH (6, 7), to represent the general range of acidity in the CF and healthy colons (43, 44); mucin (6, 8 g/L), to represent the thicker mucus layer secondary to CFTR dysfunction (3, 45); and bile salts (1, 2 g/L), to represent the impaired uptake of bile salts in CF (46, 47). We use low-CF-MiPro and median-CF-MiPro in the experiments outlined below and use MiPro as a control in all studies.

**TABLE 1:** Features and respective concentrations used in MiPro, low-CF-MiPro, and median-CF-MiPro

Feature	MiPro	Low- CF-MiPro	Median- CF-MiPro	Sources
Fat (glycerol)	0	0.5%	1%	(38, 39)
Sodium nitrate	0	0.5 mM	1 mM	(37)
Sodium sulfate	0.26 mM	0.76 mM	1.26 mM	(34, 35)
Sodium formate	0	0.5 mM	1 mM	(36)
Hydrogen peroxide	0	1 µM	10 µM	(41, 42)
Antibiotic (Bactrim)^*a*^	0	1 µM	10 µM	(40)
Mucin^*b*^	4 g/L	6 g/L	8 g/L	(3, 45)
Bile salts^*c*^	0.5 g/L	1 g/L	2 g/L	(46, 47)
pH	7	6	6	(43, 44)

^*a*^ 5:1 mix of sulfamethoxazole (Sigma-Aldrich CAS No: 723-46-6) and trimethoprim (CAS No: 738-70-5).

^*b*^ Stomach porcine mucin (Sigma-Aldrich CAS No: 84082-64-4).

^*c*^ 1:1 mix of cholic acid/deoxycholic acid (RPI CAS No: 361-09-1; 302-95-4).

### Analysis of inoculum used in these studies

To test the effects of CF-MiPro on structure and function of a given gut microbiome sample, we utilized stool samples and colonoscopy aspirates (the latter harvested from the descending colon) in the passaging experiments outlined below. These clinical samples originate from nonCF donors (*n* = 13) and CF donors (*n* = 9). Clinical sample information and preparation methods are detailed in Table 2 and Table S1, respectively. In the experiments below, we compare the pooled stool/colonoscopy data from pwCF versus nonCF controls.

**TABLE 2:** Source of stool/colonoscopy samples used in this study

Sample name	Sample type	Genotype
1.2	Stool, child	nonCF
2.1	Stool, child	nonCF
3.1	Stool, child	nonCF
4.1	Stool, child	nonCF
5.1	Stool, child	nonCF
6.1	Stool, child	nonCF
7.1	Stool, child	nonCF
8.1	Stool, child	nonCF
HV111	Colonoscopy, adult^*a*^	nonCF
HV115	Colonoscopy, adult^*a*^	nonCF
HV116	Colonoscopy, adult^*a*^	nonCF
HV117	Colonoscopy, adult^*a*^	nonCF
HV121	Colonoscopy, adult^*a*^	nonCF
110-O	Stool, child	CF
113M	Stool, child	CF
122F	Stool, child	CF
141_20210511	Stool, child	CF
143_20220510	Stool, child	CF
150_20230110	Stool, child	CF
153_20220913	Stool, child	CF
CF101	Colonoscopy, adult^*a*^	CF
CF104	Colonoscopy, adult^*a*^	CF

^*a*^ Colonoscopy aspirate from the descending colon from adults.

We determined the composition of these clinical samples using 16S rRNA gene amplicon library sequencing (Tables S1 and S2). Overall, we note a non-significant difference in average Shannon Diversity Index (SDI) values for stool/colonoscopy samples from pwCF and nonCF genotypes, as analyzed by linear regression (Fig. S1A; *P* = 0.823). SDI is an alpha diversity measure encompassing both microbial richness and evenness. In agreement with these results, we found a non-significant difference in Chao-1 index, a measure of microbial richness, for stool/colonoscopy samples from pwCF and nonCF controls (Fig. S1B *P* = 0.868).

The Bray-Curtis distance metric was used to determine the influence of genotype on microbial community similarity between samples and significance was tested with PERMANOVA. While there was modest overlap between these clinical samples from CF and nonCF sources, genotype played a significant role in beta diversity (Fig. S1C *P* = 0.001). Interestingly, there are two nonCF outliers—these samples are both colonoscopy aspirates from adult donors. We do not know whether these samples were collected at a time of inflammation or illness.

We next calculated colony forming units per milliliter (CFU/mL) of each stool or colonoscopy aspirate following growth on blood sheep agar at 0% oxygen or 21% oxygen. Total CFU/mL is the sum of CFU/mL detected ± oxygen (Fig. S1D). Of note, total CFU/mL exaggerates the number of viable bacteria since some microbes will grow ± oxygen (i.e., facultative anaerobes). We used a linear regression model to test whether genotype had a significant effect on cell count. CF samples cultured significantly less anaerobic CFU/mL (Fig. S1E; *P* = 0.0014) and aerobic CFU/mL (Fig. S1F; *P* = 0.045); thus, total CFU/mL was decreased in CF samples (Fig. S1D; *P* = 0.0017).

Lastly, we assessed broad changes in relative abundance at the phylum level between the two genotypes (Fig. S1G; Table S3). When we analyzed the clinical samples based on origin, average Proteobacteria relative abundances were 14.5% higher in CF versus nonCF samples, which is in concordance with the literature (5, 15, 19). Also, Bacteroidota (Bacteroidetes) are decreased by 14.7% in CF samples compared to nonCF samples, also consistent with the literature (5, 15, 19). Actinobacteriota (nonCF: 14%, CF: 15.6%), Firmicutes (nonCF: 47.6%, CF: 46.2%) and Verrucomicrobiota (nonCF: 0.213%, CF: 0.333%) were not significantly different between the two genotypes (Table S3).

### CF-MiPro influences alpha and beta diversity following culture *in vitro*

**Fig 1:**
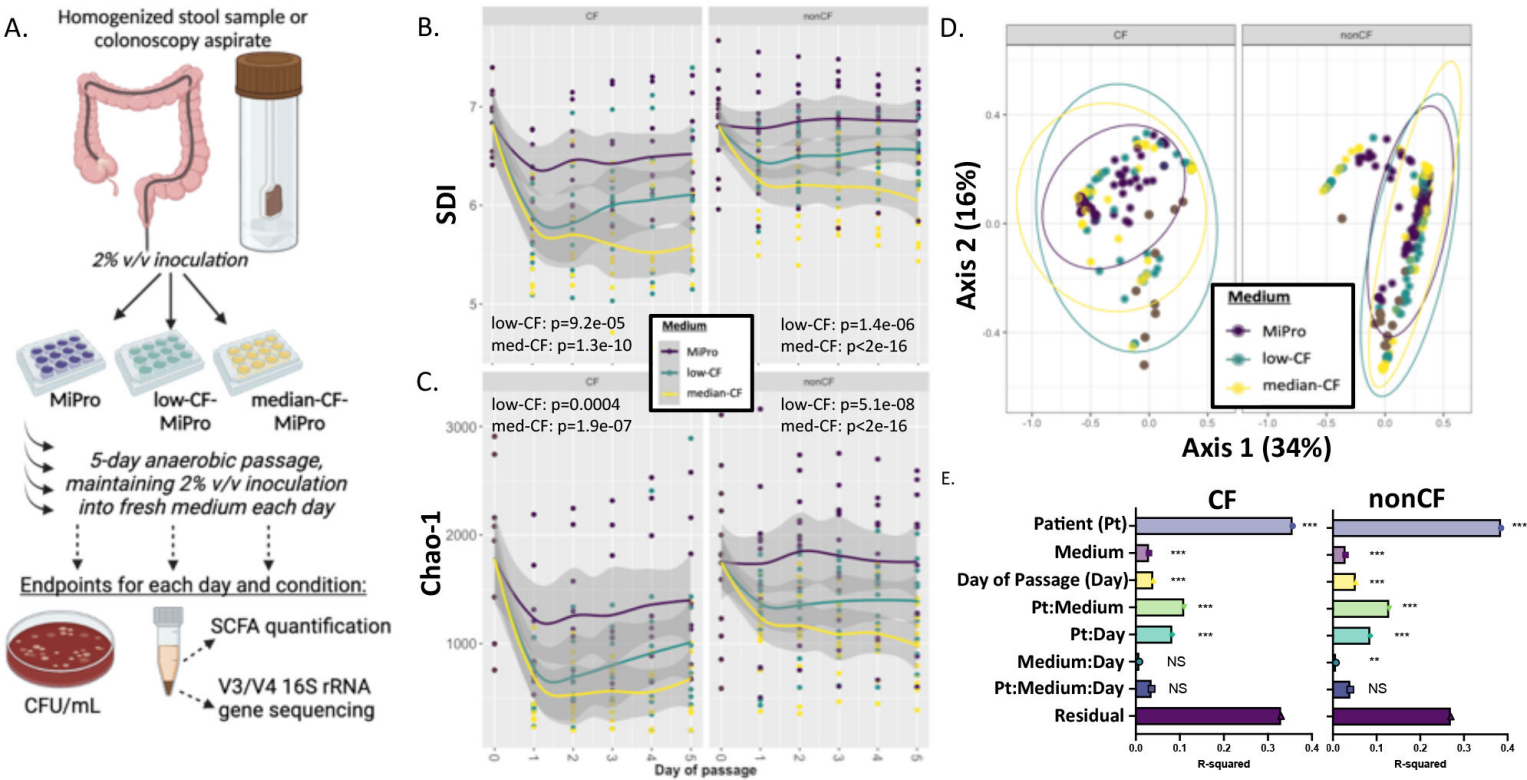
Impact of CF-MiPro on the diversity of cultured clinical samples from CF and nonCF subjects. (**A**) Schematic of the experimental design. Stool and colonoscopy samples are grown in the indicated medium over 5 days under anoxic conditions and then subjected to analysis as follows: viable counting, amplicon sequencing, and targeted analysis of SCFA. (**B**) Shannon Diversity Index (SDI) and (**C**) Chao-1 distance of CF (left) and nonCF (right) samples cultured in MiPro (purple), low-CF-MiPro (teal) and median-CF-MiPro (yellow) for 5 days. Each day, a 2% inoculum was introduced into fresh medium, with the passages every 24 h. Samples were collected at day 0 and daily though the end of day 5. A linear mixed effect model from the R package lme4 was used to test whether SDI or Chao-1 changed significantly with medium type. Patient was set as the random variable to control for multiple sampling. Both SDI and Chao-1 are significantly negatively correlated with low- and median-CF-MiPro for both genotypes, compared to MiPro. (**D**) Bray-Curtis beta diversity was calculated for each sample and displayed on a principal coordinates analysis (PCoA) plot, faceted by genotype and colored by medium. The first two components account for 50% of total variance. (**E**) Significant differences in beta diversity were tested by PERMANOVA with metadata included in the model, and R-squared values for all metadata included in model, as well as potential interactions, are plotted with significance codes indicated (NS, non-significant; **P < 0.01, ***P < 0.001). “Residual” indicates variation unexplained by the model.

To assess the impact of the media types tested here when culturing the stool and colonoscopy samples, homogenized stool or raw colonoscopy aspirates were inoculated at a 2% final volume/volume (vol/vol) ratio into MiPro, low-CF-MiPro, or median-CF-MiPro—these initial cultures are designated “day 0.” Each culture was then serially passaged anaerobically for 5 days. Each day, the cultures were homogenized by mixing, subcultured into fresh medium at 2% final inoculum, and then incubated at 37°C for 24 h before analysis. Each day, we performed the following analysis: (i) CFU/mL were calculated by plating aliquots of the homogenized, planktonic culture on sheep blood agar that were then incubated at 0% or 21% oxygen, (ii) the cell pellets were collected for DNA extraction and 16S rRNA gene amplicon library sequencing, and (iii) culture supernatants were filter-sterilized and stored for subsequent analysis of SCFA (Fig. 1A).

We generated 16S rRNA gene amplicon library sequencing data from DNA extracted from all cell pellets from all passages. We analyzed these data using a mixed linear model for each genotype with patient (or sample source) as the random effect to test whether SDI was significantly correlated with medium and day of passage. For samples originating from a CF donor, medium, and day of passage significantly correlated with decreased SDI in the CF-MiPro media formulations (Fig. 1B **,** left; low-CF-MiPro, *P* = 9.2e−05; median-CF-MiPro, *P* = 1.3e−10; day *P* = 0.0002). The same trend is observed for samples originating from a nonCF donor, whereby medium significantly correlated with decreased SDI in a dose-dependent manner (Fig. 1B, right; low-CF-MiPro, *P* = 1.4e−06; median-CF-MiPro, *P* < 2e−16; day *P* = 0.014). In fact, SDI is negatively correlated with CF-MiPro at each day of passage in both genotypes, suggesting that a shorter passage duration can replicate decreased alpha diversity (Fig. S2).

A second metric of alpha diversity, Chao-1, which estimates total richness, indicated that both formulations of CF-MiPro reduced the number of taxa in a community across stool/colonoscopy samples from pwCF (Fig. 1C, left; CF: low-CF-MiPro, *P* = 0.0004; median-CF-MiPro, *P* = 1.9e-07; day *P* = 0.0006) and from nonCF clinical samples (Fig. 1C, right; nonCF: low-CF-MiPro, *P* = 5.1e−08; median-CF-MiPro, *P* < 2e−16; day *P* = 0.001).

To confirm the use of MiPro as a maintenance medium (33), we ran a mixed linear model for each genotype with patient as the random effect to test whether SDI and/or Chao-1 were significantly correlated with day of passage. Overall, MiPro maintains SDI (Table S4, CF: day *P* = 0.293; Table S5, nonCF: day *P* = 0.411) and Chao-1 (Table S6, CF: day *P* = 0.249; Table S7, nonCF: day *P* = 0.875) measures of a given inoculum after 5 days of *in vitro* passage. Thus, our analysis replicates the findings of Li and colleagues (33) with respect to MiPro as a maintenance medium.

The Bray-Curtis distance metric was then applied to the 16S rRNA gene amplicon library sequencing data and used to test the influence of patient, medium, day of passage, and their interaction terms on overall microbiome composition within each genotype (Fig. 1D). Significance was tested by PERMANOVA (Fig. 1E). Patient, medium, and day of passage all significantly impacted community composition, with patient having the largest *R* ^2^ value. The largest shift in microbial composition of both genotypes occurs at day 1 (Fig. S3), which aligns with observed changes in alpha diversity.

We also used Jaccard and Morisita-Horn as alternative metrics of beta diversity. Jaccard index measures the similarity between groups based on occurrence measures (i.e., presence or absence of taxa) and does not consider relative abundances of taxa. Morisita-Horn index accounts for relative abundance of taxa but ignores taxa with 0% relative abundance: this metric only clusters by the presence of shared taxa, rather than both presence and absence. PCoA plots displaying both Jaccard index (Fig. S4A) and Morisita-Horn index (Fig. S4B) resemble each other and the Bray-Curtis distance (Fig. 1D). The first two components of the PCoA plot displaying Jaccard index account for 36.9% of total variance, and 60% of total variance is displayed by Morisita-Horn. For each beta diversity metric, patient contributes to the most variance for both CF and nonCF samples, followed by medium and day of passage, which contribute to similar variance between both genotypes (Fig. S4C and D). Residual values for Jaccard analysis are larger than those for Morisita-Horn, which may support the robustness of accounting for both compositionality and absent taxa.

It is notable that all combined exposures included in the model explained only 67%–73% of the total variance in beta diversity in CF and nonCF samples, respectively. The residual variance indicates that there is a modest amount of variation between samples that is not due to any of the factors examined here. To address this residual variance, we incorporated sample preparation (Prep) as an explanatory variable. Because four CF samples were initially homogenized in PBS + 10 mL l-cysteine, rather than PBS + 7.15% glycerol, we calculated the Bray-Curtis distance metric on all CF samples across all days of passage (Fig. S5). Here, we aimed to address the role of different preparation methods on microbial composition over 5 days of *in vitro* passage. Principal coordinates analysis (PCoA) plots displaying Bray-Curtis beta diversity suggest that preparation in either cysteine or glycerol results in varied microbial compositions (Figure S5A and C). To address how composition changes across days following each preparation, we ran a PERMANOVA within each “Prep” and culture condition (Medium). Results suggest that microbial composition significantly changes across all days of passage in cysteine-prepared samples, with an average *R* ^2^ value of 0.12 (Fig. S5B). Interestingly, cysteine-prepared samples passaged in median-CF-MiPro have the most variation by day (*R* ^2^ = 0.2, *P* = 0.002, Fig. S5B). Alternatively, microbial composition changes across all days of passage in glycerol-prepared samples, except for those passaged in median-CF-MiPro (*P* = 0.08, Fig. S5D). Notably, “Day of passage” contributes to less variation in composition of glycerol-prepared samples (average *R* ^2^ = 0.0474, Fig. S5D) than cysteine-prepared samples. The analyses, which incorporate all days of passage, suggest the possibility of l-cysteine-mediated selective pressure. Therefore, we suggest that preparation in glycerol may lead to less day-to-day variation when samples are subsequently cultured under these conditions.

We note four important findings from the analysis above. First, both CF and nonCF genotype significantly affect how community composition changed over days of passage (Patient:Day of passage), indicating differential rates of change within individuals, a finding consistent with the observation that “patients are most like themselves” when analyzing microbiota. Second, for nonCF samples, CF-MiPro medium significantly affected how community composition changed over days of passage (Medium:Day of passage), suggesting continuous shifts in microbial composition as the microbiota adapted to the CF-MiPro medium conditions. Furthermore, when comparing compositions of nonCF samples passaged in either low- or median-CF-MiPro, excluding MiPro-passaged samples, medium contributes significantly to differences in beta diversity (*P* = 0.007). This significance is not seen in CF samples passaged in either low- or median-CF-MiPro (*P* = 0.079), suggesting the differential role of low- and median-CF-MiPro on each genotype and the dose-dependent responses observed in nonCF samples. Third, for measures of α- and β-diversity, for both CF and nonCF samples, there was a significant interaction between patient and medium, supporting the distinct role of each individual’s response to the CF-MiPro medium. Finally, because the interaction term “Medium:Day of passage” is non-significant in altering CF community composition, we can infer that shifts in beta diversity within media types are largely static across days for this genotype. We highlight these latter two important points in the Discussion. In conclusion, these results suggest that the patient contributes the most to differences in beta diversity (independent of analytical tool used), followed by medium, method of preparation, and day of passage. Notably, the microbial composition of CF samples seems to remain relatively static over the course of passaging, while nonCF microbial compositions continue to shift over time in response to growth in CF-MiPro medium.

### CF-MiPro induces changes in relative abundance of microbiota over time to a more CF-like state

**Fig 2:**
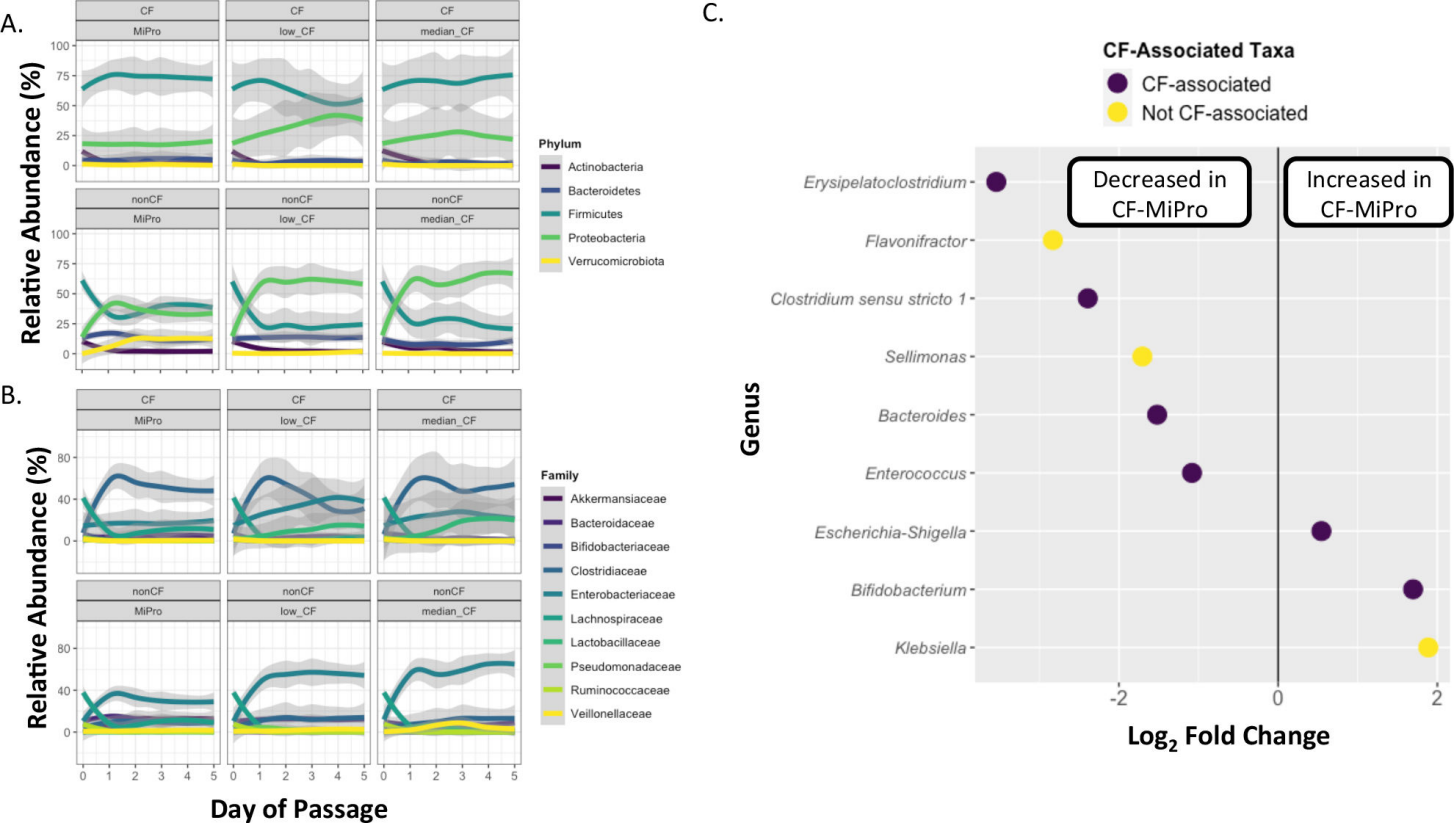
Microbial relative abundance changes across media types. Day of passage is graphed versus relative abundance of the indicated taxa for each sample, and a ribbon plot was used to visualize overall changes in microbial relative abundance at the (**A**) phylum level and (**B**) family level across all media conditions. The mean relative abundance is depicted by the line with 95% confidence interval shaded in gray. The legend indicates the taxonomic assignment for each panel. (**C**) Log_2_ fold change of taxa that were significantly (*P*-adj. < 0.05) altered in nonCF samples passaged in median-CF-MiPro versus MiPro. Taxa were filtered to be present in at least 5% of all samples. Each dot represents a single genus and is color coded by association with CF gut dysbiosis (see main text for details). Significance was determined by DESeq2 using a non-continuous model of samples binned by media condition across days of passage 1 through 5. Patient was included as a design variable to control for multiple sampling.

To understand the general shifts that occur across *in vitro* conditions, we first examined broad changes in relative abundance that occur over time at the phylum level. We compared average phylum-level relative abundances from all samples collected over the 5-day passage within each medium and genotype (Fig. 2A; Table S3). In general, phylum-level relative abundances in passaged samples from pwCF remain stable over 5 days within all three media types (Fig. 2A **, top**) although there are modest shifts in average relative abundance following *in vitro* passage in MiPro and low-CF-MiPro. For example, Firmicutes shift from an average of 63% in raw CF samples to 74.4% over the course of *in vitro* passage in MiPro, ~60% in low-CF-MiPro, and ~72% in median CF-MiPro (Table S3). Additionally, Actinobacteria decrease from 12.3% in raw CF samples to ~1.5% in all media formulations. These shifts likely occur due to the brief introduction of oxygen during sample preparation and acclimation to an *in vitro* system. The average relative abundances of the other top phyla remain generally stable between cultivation in MiPro and median-CF-MiPro, indicating that CF-MiPro can serve as a maintenance medium for CF gut microbiome samples (Table S3).

Phylum-level relative abundances of nonCF samples differ significantly from CF samples in terms of shifts in microbiota across the different media types. Shifts observed in MiPro occur at day 1 of passage in a batch-independent manner (Fig. S6), suggesting the sensitivity of nonCF gut microbiota to oxygen and/or cultivation in this medium likely due to the significantly larger concentration of detectable anaerobic microbes in nonCF stool (Fig. S1E). The largest overall changes occur in (i) Firmicutes, which decreases from an average of 62.3% in raw nonCF samples to 36.1% over the course of passage in MiPro and (ii) Proteobacteria, which increases from an average of 12.3% in raw nonCF samples to 36.9% over the course of passage in MiPro (Fig. 2A **,** bottom; Table S3). Importantly, these changes we observe in nonCF samples in MiPro are exacerbated in a dose-dependent manner in low- and median-CF-MiPro over 5 days of passage. For example, Firmicutes average relative abundance further decreases from 36.1% in MiPro to 23.7% in median-CF-MiPro (*P* = 0.003 as determined by mixed effect modeling, Table S8), while Proteobacteria increase from 36.9% in MiPro to 64.7% in median-CF-MiPro (*P* = 2.59e−09, Table S3; Table S8). Bacteroidota and Verrucomicrobiota relative abundances also change unidirectionally, with Bacteroidota decreased from 13.5% in MiPro passages to 8.32% in median-CF-MiPro passages (*P* = 5.8e−05, Table S3; Table S8), and Verrucomicrobiota reduced from 10.8% in MiPro to 0.0828% in median-CF-MiPro passages (*P* = 7.58e−11, Table S3; Table S8). These changes are consistent with comparisons of persons with CF with nonCF cohorts (5, 15, 18, 19).

Our analysis shows that in the clinical samples used here, the phyla Actinobacteria, Proteobacteria, Bacteroidota, and Verrucomicrobiota are dominated by single families: Bifidobacteriaceae, Enterobacteriaceae, Bacteroidaceae, and Akkermansiaceae, respectively. Phylum-level changes appear to be driven primarily by changes in these taxa (Fig. 2B; Table S9). In nonCF raw samples, Lachnospiraceae appears to be the dominant Firmicutes family (39.2% relative abundance, Table S9). In CF raw samples, Lachnospiraceae is also the dominant Firmicutes family, with a relative abundance of 42.9%. However, when passaged in median-CF-MiPro, Lachnospiraceae is decreased in both genotypes (CF: *P* = 0.02; nonCF: *P* = 0.0034, Table S10) as determined by mixed effect modeling with Patient as the random effect. Clostridiaceae dominates the Firmicutes family under all culture conditions in a medium-independent manner (Table S10). Furthermore, Enterobacteriaceae is enriched in nonCF cultures passaged in median-CF-MiPro compared to MiPro alone (*P* = 1.35e−10, Table S10); this enrichment is not observed in CF cultures passaged in median-CF-MiPro (*P* = 0.22, Table S10). These observations align with literature reporting the increased relative abundance of both Clostridiaceae (48) and Enterobacteriaceae (1, 5, 9, 11) and reduced Lachnospiraceae in CF stool (14).

We next determined the genera that changed significantly between media conditions across all samples. We first compared average genus-level relative abundances from all samples collected over the 5-day passage within each medium and genotype (Fig. S7; Table S11). The families Bifidobacteriaceae, Clostridiaceae, Enterobacteriaceae, Bacteroidaceae, and Akkermansiaceae are dominated by single genera: *Bifidobacteria*, *Clostridium*, *Escherichia/Shigella*, *Bacteroides,* and *Akkermansia*, respectively. Family-level changes appear to be driven primarily by changes in these taxa (Table S11). For passaged samples from pwCF, *Clostridium* is the dominant genus within the Firmicutes phylum in MiPro and CF-MiPro. In nonCF samples, *Blautia* is the dominant genus in raw, uncultured samples yet is replaced by *Clostridium* following passage in CF-MiPro.

Next, we wanted to determine which genera may be the most significantly enriched or depleted in CF-MiPro compared to MiPro. To perform this analysis, we compared a subset of samples within each genotype grouped by medium: samples cultured in MiPro and samples cultured in median-CF-MiPro. We excluded raw, uncultured samples from this analysis. We then filtered by taxa that are present at least 5% relative abundance of all samples (prevalence > 0.05) and those with relative abundances that significantly change between MiPro and median-CF-MiPro (*P*-adj. < 0.05 by Wald test statistic). Taxa that changed significantly across these conditions were compared with taxa associated with CF gut microbial dysbiosis (49). Among the nine genera that shifted significantly between media types for nonCF-passaged samples, seven genera were associated with CF gut dysbiosis. For example, *Escherichia*, specifically *E. coli*, is reported to be enriched in CF stool (5, 15, 18, 19). We found that *Escherichia/Shigella* is significantly enriched across samples passaged in median-CF-MiPro, compared to MiPro (log_2_FC = 0.54, *P*-adj. = 0.02; Fig. 2C). Additionally, SCFA-producing microbes, such as *Bacteroides* and *Akkermansia*, are reported to be depleted in pwCF. Similarly, we see a 1.52-fold decrease in *Bacteroides* in median-CF-MiPro (*P*-adj. = 2.2e−11). Of note, *Akkermansia* decreases by 1.04-fold in median-CF-MiPro among nonCF samples; however, this difference is non-significant compared to passage in MiPro (*P*-adj. = 0.52, Table S12).

Interestingly, we observed a significant decrease in two *Clostridium*-related taxa (*Erysipelatoclostridium* and *Clostridium sensu stricto 1*). There have been mixed reports of *Clostridium* relative abundances in CF (14, 21, 48, 50, 51), some of which are associated with antibiotic use (21). These results suggest intra-genus diversity among pwCF and the putative association of certain species with infection and antibiotic perturbation. Because our study lacked metadata for each patient and we used 16S rRNA gene sequencing, further investigation is needed to discern which species are depleted in median-CF-MiPro and if these species are correlated with clinical outcomes. Of note, when we performed this analysis on a subset of CF samples passaged in MiPro and median-CF-MiPro, we were not able to identify any genera that shifted in relative abundance under the same parameters used in the nonCF analysis (prevalence > 5%, *P*-adj. < 0.05, Table S13). These results support that CF-MiPro maintains CF gut microbiome samples at the genus level, relative to those passaged in MiPro, yet significantly shifts nonCF gut microbiome samples toward a “CF-like” microbial profile with less *Bacteroides* and more *Escherichia*.

To address the role of *in vitro* culture on taxonomical shifts, we repeated this analysis with the inclusion of day 0 (uncultured) samples from each genotype. Similarly, we filtered by taxa that are present at least 5% relative abundance of all samples (prevalence > 0.05) and those with relative abundances that significantly change between day 0 and day 5 in either MiPro or median-CF-MiPro (*P*-adj. < 0.05 by Wald test statistic). As suggestive of the high-level taxonomic shifts observed in nonCF samples passaged in MiPro (Fig. 2A), we identified 25 genera that were significantly enriched or depleted following 5-day passage in MiPro, compared to raw nonCF samples (Fig. S8A). Similarly, 22 genera were significantly differentially abundant following 5-day passage in MiPro, compared to raw CF samples (Fig. S8B). Of these totals, the majority of taxa were depleted in MiPro (nonCF: 17 genera; CF: 18 genera). Notably, there were nine shared genera in both genotypes that were depleted following *in vitro* culture in MiPro (e.g., *Agathobacter, Dorea, Faecalibacterium, Ruminococcus, Blautia, Anaerostipes, Collinsella, Streptococcus,* and *Bifidobacterium*); most of these taxa are SCFA-producing anaerobes. While MiPro maintains SDI and Chao-1 alpha diversity measures over the course of 5 days *in vitro* (Tables S4-7), these common depletions in anaerobic genera underscore the critical role of oxygen in this *in vitro* system. Future experimentation will incorporate preparation of raw samples in an oxygen-limiting environment.

Then, we compared relative abundances of genera that are significantly different between day 0 and day 5 samples passaged in median-CF-MiPro using the same parameters and analysis. In nonCF samples, 15 genera were differentially abundant between day 0 and day 5 in median-CF-MiPro, of which 9 were depleted (Fig. S8C). Taxa including *Faecalibacterium, Anaerostipes, Ruminococcus, Collinsella,* and *Blautia* were further depleted in median-CF-MiPro compared to MiPro based on log_2_ fold change values. Additionally, *Escherichia/Shigella* and *Enterococcus* were further enriched in median-CF-MiPro compared to MiPro. Interestingly, in CF samples, 11 genera were significantly depleted between day 0 and day 5 in median-CF-MiPro and 0 genera were enriched (Fig. S8D). Among those depleted, *Dorea, Blautia, Erysipelatoclostridium, Streptococcus, Ruminococcus, Collinsella, Bifidobacterium,* and *Bacteroides* are further depleted in median-CF-MiPro compared to MiPro, based on log_2_ fold change values. These results suggest that median-CF-MiPro drives a CF-like microbial composition in nonCF gut microbiome samples and may exacerbate existing microbial dysbiosis in some CF samples.

Overall, these results highlight: (i) the highest observed change in taxa abundance occurs when nonCF samples are passaged in CF-MiPro medium, and these changes are larger for median-CF-MiPro versus low-CF-MiPro, indicating a “dose-dependent” response, (ii) the overall patterns of change appear to be driven by a small number of bacterial families, and often a sole family, (iii) the shifts observed in CF-MiPro largely align with clinical observations of the shifts in phyla and genera associated with pwCF, and importantly, (iv) for nonCF samples, passaging in CF-MiPro results in a shift in these samples to a CF-like microbiota composition.

### CF-MiPro decreases absolute microbial abundance

**Fig 3:**
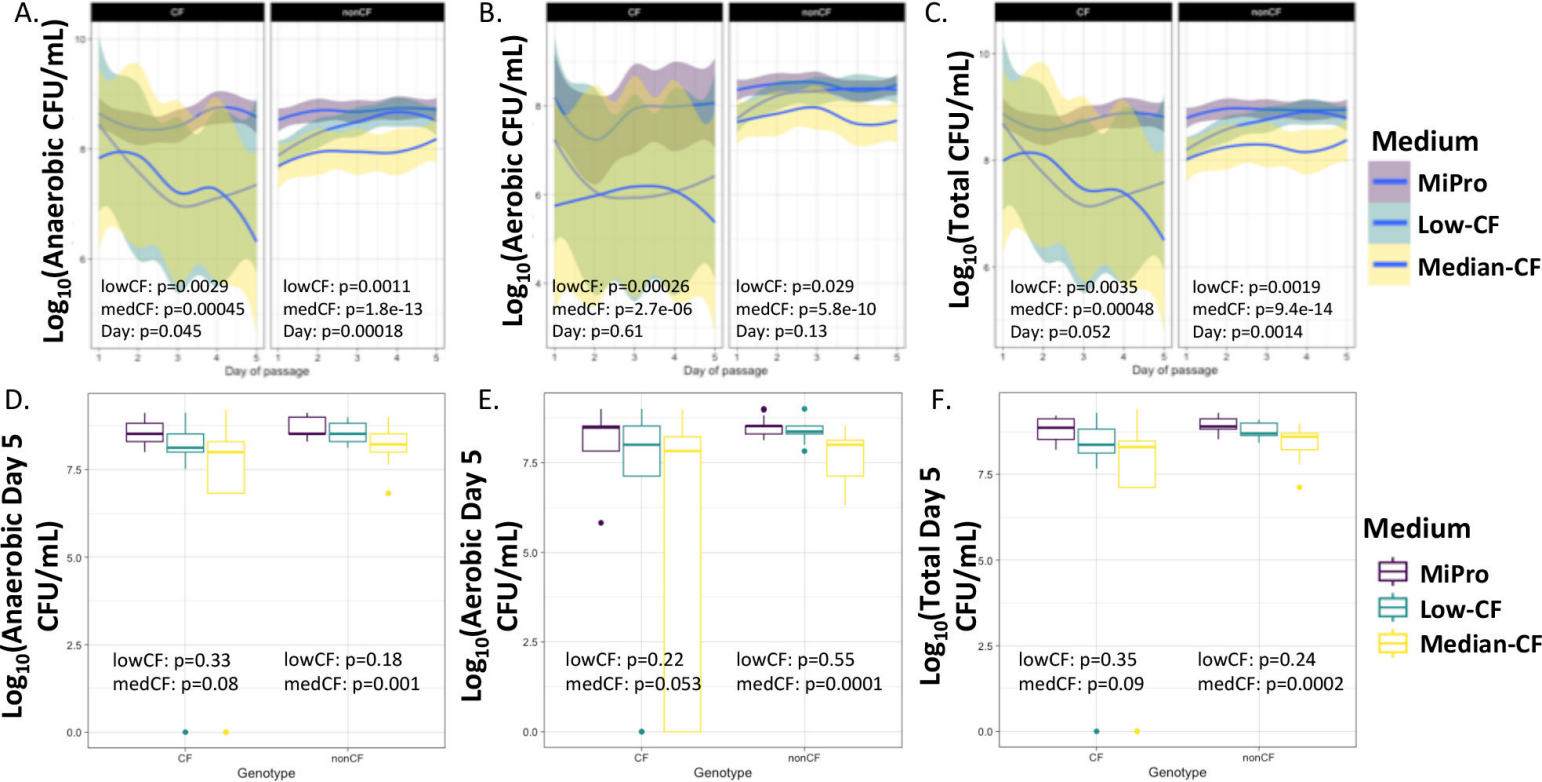
CFU/mL of nonCF and CF stool or colonoscopy samples passaged in CF-MiPro over five days. NonCF (*n* = 13) and CF (*n* = 9) stool and colonoscopy samples were inoculated into each medium (MiPro, purple; low-CF-MiPro, teal; median-CF-MiPro, yellow) at a 2% final (vol/vol) ratio and passaged for 5 days, using this 2% inoculum into fresh medium daily. Each day, viable cell counts were quantified on sheep blood agar plates incubated at (**A**) 0% oxygen for obligate anaerobe and facultative anaerobe detection or (**B**) 21% oxygen for aerobic/facultative anaerobe detection. Total CFU/mL [log_10_(aerobic + anaerobic CFU/mL)] are displayed in (**C**). A linear mixed effect model from the R package lme4 was used to test whether CFU/mL within each genotype changed significantly with medium type and day of passage. Patient was set as the random variable to control for multiple sampling. CFU/mL are typically negatively correlated with CF-MiPro, in a dose-dependent manner, for the cultured CF and nonCF stool and colonoscopy samples, suggesting a decrease in richness. Overall, day of passage is positively correlated with CFU/mL for CF and nonCF cultured samples. To eliminate the contribution of Day of Passage, (**D**) anaerobic, (**E**) aerobic, and (**F**) total CFU/mL from day 5 are graphed by genotype, colored by medium. Another linear mixed effect model, with patient set as the random variable, was used to test whether day 5 CFU/mL within each genotype changed significantly with medium. Under both oxygen exposures, CFU/mL from CF samples used as inoculum were not correlated with medium, while CFU/mL from nonCF samples used as inoculum were negatively correlated with median-CF-MiPro but not low-CF-MiPro.

We next examined CFU/mL of each sample as a read-out of the culturable, absolute abundance of the microbial population. Inocula (raw samples) and *in vitro* passaged cultures were serially diluted 10-fold and plated on blood sheep agar and then incubated at either 0% or 21% oxygen for 24 h. We used a mixed effect linear model for each genotype with patient as the random effect to test whether CFU/mL was significantly correlated with medium or day of passage. As previously described, raw CF samples cultured less aerobic and anaerobic CFU/mL (Fig. S1D through F). In conjunction with decreased richness observed in samples cultured in CF-MiPro (Fig. 1C), it was our expectation that CF-MiPro would foster fewer total microbes. Across clinical samples from both genotypes cultured through MiPro, low-CF-MiPro and median-CF-MiPro for 5 days, CF-MiPro showed decreased anaerobic CFU/mL in a dose-dependent manner (Fig. 3A). This decrease in anaerobic CFU/mL in CF-MiPro is day-dependent in nonCF cultures (day, *P* = 0.00018) and in CF cultures (day, *P* = 0.045). Similarly, the aerobic CFU/mL fraction is reduced over passages in all media; although, this reduction is observed in a day-independent manner for both genotypes and the reductions are more modest (Fig. 3B).

CF-MiPro is significantly correlated with decreased total CFU/mL (sum of aerobic and anaerobic CFU, then log_10_-transformed) for clinical samples from both subject genotypes, in a day-dependent manner for nonCF samples (day, *P* = 0.0014) and marginally significant in CF samples (day, *P* = 0.052). To account for day-to-day variability, we next graphed CFU/mL calculated from Day of Passage 5 from both oxygen exposures. Linear regression, with Medium as the explanatory variable, was used for statistical analysis. Anaerobic CFU/mL at day 5 are not significantly affected by medium in cultures inoculated with CF samples (Fig. 3D, left; low-CF-MiPro, *P* = 0.33; median-CF-MiPro *P* = 0.08), but median-CF-MiPro significantly decreases day 5 anaerobic CFU/mL in cultures inoculated with nonCF samples (Fig. 3D, right; low-CF-MiPro, *P* = 0.18; median-CF-MiPro, *P* = 0.001). This observation is repeated for the aerobic day 5 CFU/mL, whereby only cultures inoculated with nonCF samples are decreased in median-CF-MiPro (Fig. 3E). Decreased total CFU/mL in cultures inoculated with nonCF samples, but not CF samples, are correlated with median-CF-MiPro (Fig. 3F). Taken together, these results suggest that samples from both genotypes are affected by CF-MiPro, as evident by the trend of decreasing CFU/mL in low- and median-CF-MiPro, but only nonCF samples are statistically significant, likely due to the greater variability within CF samples. The variability among CF samples, especially those passaged in median-CF-MiPro, may indicate that median-CF-MiPro can further exacerbate dysbiosis of select CF samples. Further experimentation is needed to identify characteristics or signatures of CF gut microbiome samples that may be more susceptible to change in CF-MiPro.

### CF-MiPro shifts short-chain fatty acid profiles

Short-chain fatty acids are metabolic byproducts of dietary fiber catabolism (49). These metabolites are the preferred nutritional source for host enterocytes and are known to have antimicrobial and anti-inflammatory properties (52). Two studies have reported a significant reduction in SCFA concentrations in CF stool compared to nonCF stool (48, 51), an observation that is likely due to the reduction in SCFA-producing microbes (53) and/or the enrichment of KEGG-annotated SCFA catabolism genes in CF stool (16). It was our prediction that, given the shift to a more CF-like microbiota in CF-MiPro, passages in this medium would lead to a decrease in SCFA concentrations over the course of 5 days of passaging.

To analyze the impact of medium on SCFA production, filtered supernatants from all cultured samples (nonCF, *n* = 12; CF, *n* = 8) collected through days 1–5 and select stool samples (“day 0”: nonCF, *n* = 8; CF, *n* = 7) were analyzed using gas chromatography-mass spectrometry (GC/MS). SCFA concentrations were determined by normalization to standards, with a detection limit of 0.3 µM. Then, these concentrations were normalized by baseline levels detected in each respective medium (MiPro, low-CF-MiPro and median-CF-MiPro). Finally, these concentrations were normalized to the CFU/mL calculated in that exact sample to account for differences in microbial density. We used a mixed linear model for each genotype with patient as the random effect to test whether each SCFA measured (acetate, propionate, butyrate) was significantly correlated with medium or day of passage.

**Fig 4:**
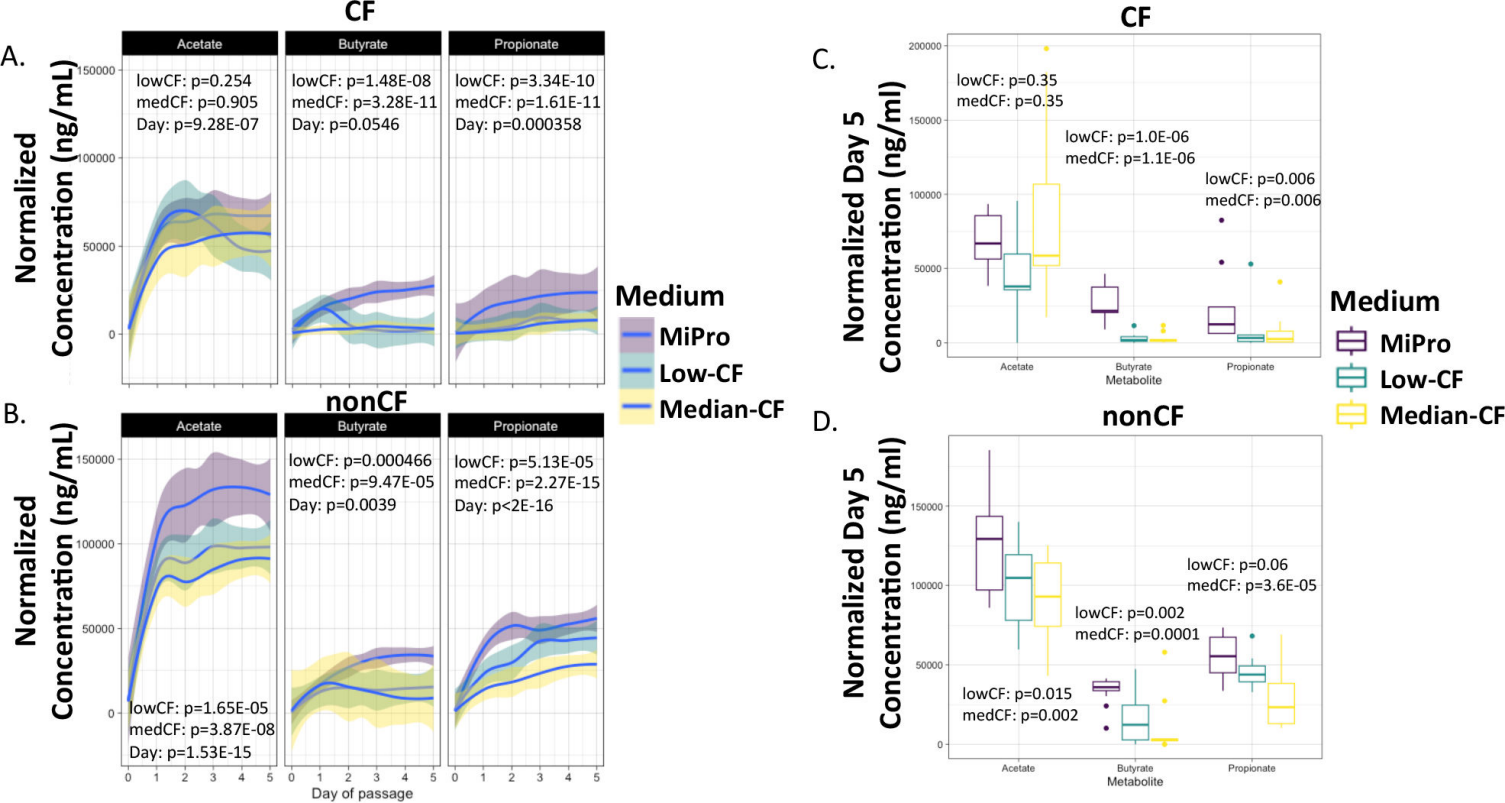
Short-chain fatty acid (SCFA) production is decreased in CF-MiPro. Raw stool samples (day 0) and filtered supernatants from days 1 to 5 cultures were analyzed via GC/MS for quantification of the three most abundant SCFA: acetate, butyrate, and propionate. The final concentrations were normalized by stool weight (day 0) or to total anaerobic CFU/mL (days 1–5) and graphed against Day of Passage for (**A**) CF and (**B**) nonCF samples. A linear mixed effect model from the R package lme4 was used to test whether each SCFA concentration within each genotype changed significantly with medium type and day of passage. Patient was set as the random variable to control for multiple sampling. While acetate concentrations were unaffected, butyrate and propionate concentrations were negatively correlated with CF-MiPro when CF samples were used as inoculum. In contrast, all three SCFA were negatively correlated with CF-MiPro when nonCF samples were used as inoculum. Day of passage inconsistently had a positive correlation with SCFA concentrations. To eliminate the contribution of Day of Passage, concentrations of each SCFA from day 5 are plotted for (**C**) CF samples and (**D**) nonCF samples. Statistical analysis was performed using a similar linear mixed effect model as in (**A**) and (**B**). At day 5, butyrate and propionate concentrations are negatively correlated with CF-MiPro across both genotypes of clinical samples.

First, SCFA concentrations from stool collected from children with and without CF do not significantly vary by genotype when analyzed using linear regression (Fig. S9). Interestingly, SCFA concentrations of acetate, butyrate, and propionate trend higher in stool from CF children, yet with great variability. There appear to be two outliers in the CF cohort which contribute to variability; this may be due to age. Further experimentation is needed to address how age determines SCFA concentration in this cohort. Regardless of genotype, levels of acetate, butyrate, and propionate are significantly lower in raw stool compared to cultured conditions. For samples from pwCF, acetate is not significantly correlated with medium. At day 1, acetate levels increase significantly; however, changes between days 1–5 are nonsignificant (Fig. 4A *,* left). Butyrate and propionate concentrations are largely maintained in CF-MiPro relative to day 0 CF samples. MiPro facilitates the growth of existing microbes and SCFA-producers, thus butyrate and propionate concentrations increase in MiPro with Day of Passage (Fig. 4A, middle and right). In nonCF samples, all three SCFA concentrations are negatively correlated with CF-MiPro and positively correlated with day, as SCFA concentrations significantly increase at day 1. Between days 1 and 5, only propionate concentrations positively correlated with day (Fig. 4B). These findings indicate that CF-MiPro supports lesser SCFA production than MiPro in both genotypes, with butyrate and propionate production mimicking that of raw CF stool.

To account for day-to-day variability, we next graphed SCFA concentrations from Day of Passage 5 for each genotype. Linear regression, with medium as the explanatory variable, was used for statistical analysis. In cultures inoculated with CF samples, acetate concentrations remain unaffected by medium, and butyrate and propionate concentrations are both significantly reduced in low- and median-CF-MiPro (Fig. 4C). In nonCF samples, acetate, butyrate, and propionate concentrations are significantly reduced in CF-MiPro in a dose-dependent manner (Fig. 4D).

**Fig 5:**
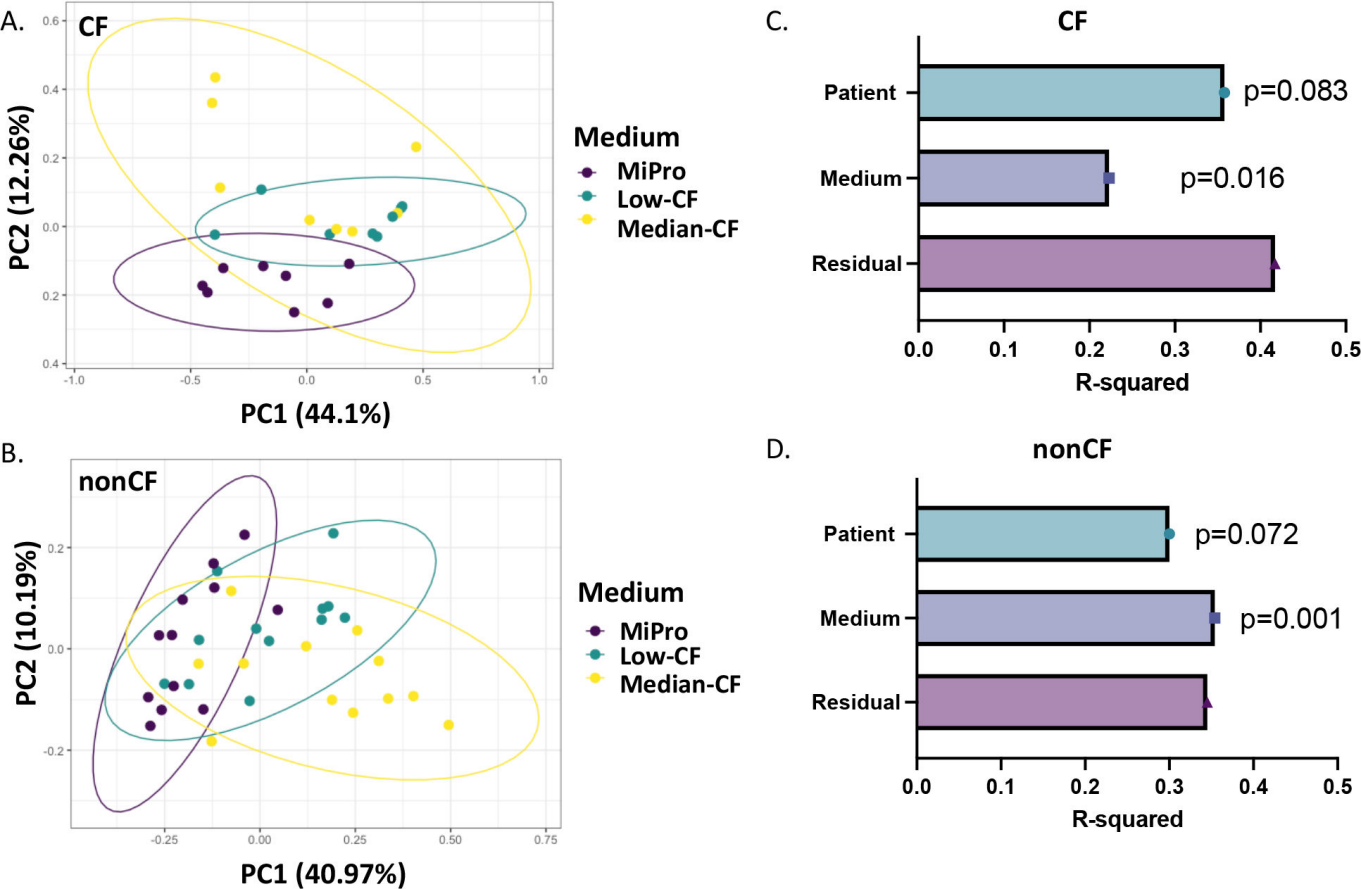
Short-chain fatty acid (SCFA) profiles are distinguished by medium in nonCF samples. CFU-normalized concentrations of acetate, butyrate, and propionate at day 5 of passage were used to calculate Bray-Curtis beta diversity for (**A**) CF and (**B**) nonCF stool and colonoscopy samples. Ordination of each genotype was displayed in principal coordinates analysis (PCoA) plots, colored by medium. For CF-derived samples, the first two components account for 56.36% of total variance. For nonCF-derived samples, the first two components account for 51.16% of total variance. Significant differences in beta diversity for (**C**) CF and (**D**) nonCF samples were tested by PERMANOVA with metadata included in the model, and *R*-squared values for Medium and Patient are plotted with respective *P*-values. “Residual” indicates variation unexplained by the model. SCFA profiles in CF and nonCF samples are significantly distinguished by medium in a patient-independent manner, yet the effect size (***R* ^2^**) is greater and in the nonCF samples.

We next examined how overall changes to the SCFA levels, including normalized concentrations of acetate, butyrate, and propionate changed across media conditions at day 5 of passage. Here, we used the Bray-Curtis distance metric to test the influence of patient and medium on overall SCFA composition in CF samples (Fig. 5A) and nonCF samples (Fig. 5B). Significance was tested by PERMANOVA within each genotype.

For samples from pwCF as the inoculum, the first two components of the PCoA plot accounted for 56.36% of total variance (Fig. 5A), with patient having the largest *R* ^2^ value, with a nonsignificant correlation with beta diversity (Fig. 5C, *R* ^2^ = 0.36, *P* = 0.083). Medium significantly correlated with beta diversity (Fig. 5C, *R* ^2^ = 0.22, *P* = 0.016) for samples from pwCF in concordance with significant decreases in normalized SCFA concentrations (Fig. 4A and C).

For nonCF samples used as the inoculum, the first two components of the PCoA plot accounted for 51.16% of total variance (Fig. 5B), with medium having the largest *R* ^2^ value, with a significant correlation with beta diversity (Fig. 5D, *R* ^2^ = 0.354, *P* = 0.001). Patient contributed non-significantly to SCFA composition (Fig. 5D, *R* ^2^ = 0.3, *P* = 0.072). Thus, nonCF SCFA profiles are significantly influenced by medium, in a patient-independent manner. Both exposures included in the model (patient and medium) explained only 59%–65% of the total variance in beta diversity in CF and nonCF samples, respectively. The residual variance indicates that there is a modest amount of variation between samples that is not due to any of the factors examined here.

Overall, these results suggest that CF-MiPro is significantly correlated with reduced levels of SCFA, with a larger effect size when nonCF samples are used as inoculum versus samples from pwCF. In contrast, for samples passaged in MiPro at Days 1-5, there is a non-significant change in SCFA regardless of inoculum, consistent with the reports that MiPro can maintain gut microbiota from clinical sources *in vitro*.

## DISCUSSION

In this study, we developed a novel first-generation *in vitro* culture medium, called CF-MiPro, to mimic the physiological conditions of the CF colon. Similar to the early approaches used in the generation of Artificial Sputum Medium [ASM (54, 55)], a culture medium used to mimic the nutritional conditions of the CF airway, we incorporated validation of CF-MiPro as part of our analysis. We adopted a previously used experimental approach (33) to track community structure (utilizing 16S rRNA gene amplicon sequencing) and function (SCFA quantification) of 22 total gut microbiome samples over time for these media.

First, our results highlight that the composition of CF gut microbiome samples is generally maintained in CF-MiPro, relative to MiPro. In low- and median-CF-MiPro, we observe similar trends, whereby CF gut microbiome samples stabilize with similar relative abundances of the top five phyla. In fact, no genera were identified to be significantly enriched or depleted in median-CF-MiPro when stool or colonoscopy samples from pwCF were passaged. Beta-diversity analysis of community composition results in a non-significant interaction between medium and day of passage in CF-derived samples, suggesting that day of passage does not alter microbial composition for CF-MiPro medium. Thus, composition of CF samples remains largely stable over the 5 days of passage when grown in CF-MiPro.

Conversely, nonCF gut microbiome samples undergo significant taxonomical shifts across all media. MiPro significantly alters 25 genera across all days of passage although these shifts largely occur at day 1 with depletions of anaerobes. Alterations in taxa occur consistently across all nonCF samples, regardless of sample type (stool or colonoscopy aspirate) or sequencing batch. These results support oxygen as a mechanism of composition alteration. NonCF gut microbiome samples experience more robust taxonomical shifts when introduced to CF-MiPro, in a dose-dependent manner. That is, passage of these nonCF stool or colonoscopy samples as inocula in low-CF-MiPro induces taxonomical shifts that are exacerbated in median-CF-MiPro. Our results highlight that the highest-abundance taxa change with medium, with overall patterns being driven by a small number of bacterial families and genera. These shifts observed in CF-MiPro generally align with clinical observations associated with pwCF. For example, analysis of stool from cwCF across cohorts, both nationally and globally, consistently reports an increase in Proteobacteria, largely driven by *Escherichia coli* (5, 15, 18, 56). When nonCF samples are passaged in CF-MiPro, *E. coli* dominates the community after 24 h. Over the course of a 5-day passage, *Escherichia-Shigella* is significantly enriched in median-CF-MiPro. A key clinically relevant microbe that is reported to be depleted in the CF gut, *Bacteroides*, is observed to be significantly depleted in median-CF-MiPro over the duration of *in vitro* passaging. These results suggest that, for nonCF samples, passaging in CF-MiPro promotes a shift to a CF-like microbiota composition. Furthermore, beta-diversity analysis of nonCF-derived samples as inocula result in a significant interaction between medium and day of passage, suggesting that day alters community composition within each medium. That is, the communities become more “CF-like” over time, as indicated through shifts in CF-associated taxa, and the gut microbiota of these nonCF samples is dynamically shifting over the 5-day duration, thus presumably continuously responding to one or more components in CF-MiPro. Furthermore, when we compare compositions of nonCF samples passaged only in low- or median-CF-MiPro, medium plays a significant role in beta diversity, suggesting that microbiomes from nonCF samples respond differently to various doses of CF-MiPro.

For both CF and nonCF samples cultivated in each medium type, we report a significant interaction between patient and medium. This result underscores the important role of person-to-person variation in response to different stimuli, in this case medium. Furthermore, in every analysis, patient is reported to have the largest *R*-squared value, indicating the largest contribution to variance. These results emphasize the need to account for systematic differences between patients in all statistical approaches. Here, we account for patients through mixed effect models by setting patient as the random variable.

In our functional analysis, which serves as a second means to validate the utility of CF-MiPro, we tested SCFA concentrations in cultivated samples. We report a significant dose-dependent depletion of butyrate and propionate in CF-MiPro, compared to MiPro. This depletion is observed in both CF and nonCF samples and aligns with the reduction in SCFA-producing microbes, such as *Bacteroides*. However, when comparing the overall SCFA composition (inclusive of acetate, butyrate, and propionate concentrations) at day 5 via Bray-Curtis dissimilarity, medium has a more significant effect on SCFA profiles in nonCF samples than CF samples, represented through an increased R-squared value. In fact, medium significantly contributes to nonCF SCFA concentrations in a patient-independent manner. These results support the overall maintenance of function for samples derived from pwCF in CF-MiPro, as tested through one functional output: SCFA quantification. As a control, we show that MiPro, as reported previously (33), maintains the population composition and permits expansion of SCFA concentrations over the course of the experiment.

Of note, sample preparation (in either l-cysteine or glycerol) contributed to significant variance in microbial composition among CF samples. It was our hypothesis that samples prepared in l-cysteine, a reducing agent, would maintain composition and experience less variance at day 1, compared to samples prepared in glycerol. Yet, Bray-Curtis dissimilarity and PERMANOVA analyses suggest otherwise: CF samples prepared in glycerol exhibit less day-to-day variation across all media types compared to those prepared in l-cysteine. These results suggest that l-cysteine may drive some level of selective pressure, especially under conditions similar to median-CF-MiPro.

Overall, we view this first-generation CF-MiPro formulation as another tool, along with *in vitro* tissue culture/organoid and animal models, to better understand the microbiota dysbiosis associated with CF. Despite the ability of CF-MiPro medium to maintain compositional and functional features of CF microbial communities derived from the gut, we note a limitation of CF-MiPro: it uses non-physiological substrates in its formulation. The source of “fat” in CF-MiPro is glycerol; the actual *in vivo* source of fat is triglycerides and their breakdown products (15, 57). Similarly, the source of sugars is from yeast extract (i.e., mannose from the yeast cell wall, glucose, and glycogen as intracellular carbohydrate sources) (33, 58) rather than the complex carbohydrates typically found in the gut (i.e., oligosaccharides and fibers) (59). Other features, like bile, are poorly characterized (or not investigated at all) in young CF cohorts. Future work will focus on modifying features of CF-MiPro as new data emerge describing the intestinal environment. This iterative process will help us to better reflect the CF intestinal environment *in vitro* and identify key features associated with microbial dysbiosis.

## MATERIALS AND METHODS

### Samples, culture conditions, and sequencing

A total of 22 samples (13 nonCF, 9 CF) were used for these experiments. Among the 13 nonCF samples, 8 were from a raw stool sample collected from children (approximate age <18–36 months), and 5 were from a colonoscopy aspirate from the descending colon of adults. Among the 9 CF samples, 7 were from a raw stool sample collected from children (approximate age <18–36 months), and 2 were from a colonoscopy aspirate from the descending colon of adults. See Table 2 for details. The stool samples were thawed, weighed, and resuspended in sterile PBS (Corning Cat. #21-040-CM) supplemented with 7.15% glycerol (and for some samples with l-cysteine added at 10 mM, see Fig. S2) in a ratio of 1:5.7(wt/vol) under aerobic conditions. The colonoscopy aspirates were frozen without processing, or without addition of glycerol.

For the passaging experiments, each sample was homogenized and inoculated into MiPro, low-CF-MiPro, and median-CF-MiPro, which were acclimatized to anaerobic conditions >24 h prior to experimentation, in a sterile 12-well plate (Corning Cat. #3512) to a final (vol/vol) ratio of 2%—this culture is designated “day 0.” Each culture was passaged anaerobically for 5 consecutive days (24 h per passage) in either a GasPak jar or an anaerobic chamber. Each day, the culture was homogenized by mixing and sub-cultured into fresh media at a 2% final (vol/vol) ratio. The remaining culture was analyzed as follows: (i) CFU/mL were calculated by plating aliquot of the planktonic culture on sheep blood agar that was then incubated at 0% or 21% oxygen, (ii) the cell pellets were collected and stored in QIAGEN RNAprotect (Cat.# 76506) for DNA extraction and 16S rRNA gene amplicon library sequencing. Homogenized stool samples or colonoscopy aspirates, as well as cell pellets from each day of passage, were stored at −80°C and later processed with Zymo fecal DNA miniprep kit (Cat.# D6010). Paired-end reads were generated with 2 × 301 Illumina NextSeq2000 amplicon sequencing of the V3-V4 hypervariable region of the 16S rRNA gene (SeqCenter, LLC. Pittsburgh, PA). Raw data have been uploaded to the NCBI sequence read archive under BioProject PRJNA999361, and (iii) culture supernatants were filter-sterilized and stored at −80°C for subsequent analysis of short-chain fatty acids (SCFA).

### Processing of rRNA-encoding gene amplicons

Primer sequences were removed by CUTADAPT (version 1.18). All subsequent preprocessing steps were performed in R version 4.2.2. The code is available at https://github.com/GeiselBiofilm/. A total of 61,916,801 raw paired-end reads were filtered and trimmed with DADA2 version 1.24.0. Reads were then denoised, merged, and chimeras removed. The final counts were 23,804,538 total reads; 60,112 mean reads per sample; and 42,103 unique amplicon sequence variants (ASVs). Taxonomy was assigned with DADA2 and the Silva version 138.1 training set. ASV taxonomy tables and sample metadata are available in supplemental tables (Table S1 and S2). ASV counts for each sample are available at https://github.com/GeiselBiofilm.

### Analysis of rRNA-encoding gene amplicons

All downstream analysis and visualization were performed in R (version 4.2.2) Phyloseq (version 1.40) and ggplot2 (version 3.4.2) were used for data handling and visualization unless otherwise noted. Reads per sample were graphed and filtered to include only samples with >9,500 reads. All samples had at least 9,500 reads following processing and were, thus, included. Linear mixed models were used for statistical regression analysis (lme4 version 1.1.34). For each sample, beta diversity was calculated by Bray-Curtis, Morisita-Horn, and Jaccard distances, and multidimensional scaling ordination was performed. Significant differences in beta diversity were tested by permutational analysis of variance (PERMANOVA) (vegan version 2.6.4).

### Metabolite quantification and analysis

Stool extracts and undiluted, filter-sterilized supernatants from day 1 to 5 cultures were stored frozen at −80°C prior to metabolite quantification. Frozen samples were shipped to Michigan State University Mass Spectrometry and Metabolomics Core (MSU MSMC, East Lansing, MI; protocol ID: MSU_MSMC_010a) for analysis by gas chromatography/mass spectrometry (GC/MS). SCFA concentrations were calculated by normalization to standards and then normalized by baseline levels detected in each media (MiPro, low-CF-MiPro, and median-CF-MiPro). Finally, these concentrations were normalized to either the weight of stool (for day 0 samples) or to the CFU/mL calculated in that exact sample (for cultured samples) to account for differences in microbial density. SCFA concentrations over time were displayed using ggplot2 (version 3.4.2), and linear mixed models were used for statistical regression analysis to account for repeated measures within sample source (lme4 version 1.1.34). For each sample, beta diversity was calculated by Bray-Curtis distance, and multidimensional scaling ordination was performed via PCoA plots. Significant differences in beta diversity were tested by permutational analysis of variance (PERMANOVA) (vegan version 2.6.4).

## SUPPLEMENTAL MATERIAL

Supplemental tables jb.00286-23-s0001.pdf Tables S3 to S11.

Supplemental figures jb.00286-23-s0002.pdf Fig. S1 to S9.

Supplemental methods jb.00286-23-s0003.pdf Protocol: in vitro passaging with CF-MiPro.

Table S1 jb.00286-23-s0004.xlsx CFMeta data.

Table S2 jb.00286-23-s0005.xlsx ASVs and annotated taxonomy.

Table S12 jb.00286-23-s0006.xlsx Differentially abundant genera from nonCF samples passaged in median-CF-MiPro and MiPro.

Table S13 jb.00286-23-s0007.xlsx Differentially abundant genera from CF samples passaged in median-CF-MiPro and MiPro.

ASM does not own the copyrights to Supplemental Material that may be linked to, or accessed through, an article. The authors have granted ASM a non-exclusive, world-wide license to publish the Supplemental Material files. Please contact the corresponding author directly for reuse.
