# Supplementary material for: An in vitro medium for modeling gut dysbiosis associated with cystic fibrosis
Source: J Bacteriol. 2024 Jan 3;206(1):e00286-23. doi: 10.1128/jb.00286-23 (PMC10810206; doi:10.1128/jb.00286-23)
Supplement: Supplemental tables — Tables S3 to S11. [file jb.00286-23-s0001.pdf]

Supplemental Tables

Table S3. Relative abundance of major phyla across media conditions.

Phylum	CF				nonCF			
	Raw*	MiPro**	Low-CF-MiPro	Median-CF-MiPro	Raw	MiPro	Low-CF-MiPro	Median-CF-MiPro
**Actinobacteria**	12.3	1.47	1.85	1.73	10.5	2.03	2.41	2.93
**Bacteroidota**	4.89	5.19	2.93	1.86	12.7	13.5	13.5	8.32
**Firmicutes**	63	74.4	60.1	72.2	62.3	36.1	22.2	23.7
**Proteobacteria**	18.3	18.3	35.1	24.2	12.3	36.9	60.8	64.7
**Verrucomicrobiota**	1.27	0.543	0.0088	0.0113	0.441	10.8	0.727	0.0828

*"Raw" represents the average relative abundance of the indicated phylum in uncultured colonoscopy aspirate or homogenized stool.
**Each medium condition represents the average relative abundance of the indicated phylum across all days of passage (1-5) in that medium.

Table S4. Mixed linear model of SDI from CF samples cultured in MiPro over five days.

	Shannon		
Predictors	*Estimates*	*CI*	*p*
(Intercept)	6.58	6.28 – 6.88	**<0.001**
Day of passage	-0.03	-0.08 – 0.03	0.293
**Random Effects**			
σ^2	0.11		
$\tau_{00\ Patient}$	0.14		
ICC	0.55		
$N_{Patient}$	9		
Observations	54		
Marginal R^2 / Conditional R^2	0.009 / 0.558		

Table S5. Mixed linear model of SDI from nonCF samples cultured in MiPro over five days.

	Shannon		
Predictors	*Estimates*	*CI*	*p*
(Intercept)	6.80	6.58 – 7.03	**<0.001**
Day of passage	0.01	-0.02 – 0.04	0.411
**Random Effects**			
σ^2	0.05		
τ_{00} Patient	0.14		
ICC	0.73		
N Patient	13		
Observations	78		
Marginal R^2 / Conditional R^2	0.002 / 0.731		

Table S6. Mixed linear model of Chao1 from CF samples cultured in MiPro over five days.

	Chao 1		
Predictors	*Estimates*	*CI*	*p*
(Intercept)	1464.40	1041.55 – 1887.24	**<0.001**
Day of passage	-37.31	-101.63 – 27.00	0.249
**Random Effects**			
σ^2	161480.77		
$\tau_{00\ Patient}$	314292.17		
ICC	0.66		
$N_{Patient}$	9		
Observations	54		
Marginal R^2 / Conditional R^2	0.009 / 0.664		

Table S7. Mixed linear model of Chao1 from nonCF samples culture in MiPro over five days.

	Chao 1		
Predictors	*Estimates*	*CI*	*p*
(Intercept)	1761.01	1416.53 – 2105.49	**<0.001**
Day of passage	2.85	-33.17 – 38.87	0.875
**Random Effects**			
σ^2	74339.07		
$\tau_{00\ Patient}$	349621.92		
ICC	0.82		
$N_{Patient}$	13		
Observations	78		
Marginal R^2 / Conditional R^2	0.000 / 0.825		

Table S8. Statistical analyses of relative abundance of the top five phyla as determined by mixed-effect linear modeling with Patient set as random effect.

Phylum	Genotype	Fixed effect	t-value	p-value	Significance code*
**Actinobacteria**	CF	Medium: low-CF	0.29	0.78	NS
		Medium: med-CF	0.20	0.84	NS
		Day	-5.80	3.74e-08	***
	nonCF	Medium: low-CF	0.38	0.71	NS
		Medium: med-CF	0.89	0.37	NS
		Day	-6.94	4.36e-11	***
**Bacteroidota**	CF	Medium: low-CF	-2.34	0.02	*
		Medium: med-CF	-3.45	0.0007	***
		Day	0.057	0.95	NS
	nonCF	Medium: low-CF	-0.021	0.98	NS
		Medium: med-CF	-4.1	5.8e-05	***
		Day	-1.12	0.26	NS
**Firmicutes**	CF	Medium: low-CF	-2.96	0.0036	**
		Medium: med-CF	-0.46	0.64	NS
		Day	-0.12	0.90	NS
	nonCF	Medium: low-CF	-3.37	0.00089	***
		Medium: med-CF	-3.01	0.0030	**
		Day	-5.33	2.45e-07	***
**Proteobacteria**	CF	Medium: low-CF	3.33	0.0011	**
		Medium: med-CF	1.17	0.24	NS
		Day	1.82	0.071	NS
	nonCF	Medium: low-CF	5.34	2.30e-07	***
		Medium: med-CF	6.21	2.59e-09	***
		Day	5.92	1.21e-08	***
**Verruco-microbiota**	CF	Medium: low-CF	-1.61	0.11	NS
		Medium: med-CF	-1.6	0.11	NS
		Day	-2.5	0.013	*
	nonCF	Medium: low-CF	-6.43	7.78e-10	***
		Medium: med-CF	-6.85	7.58e-11	***
		Day	2.99	0.0031	**

*Significant codes indicate: (NS: non-significant, *: p<0.05, **: p<0.01, ***: p<0.001)

Table S9. Relative abundance of top 10 families across media conditions.

Family	CF				nonCF			
	Raw*	MiPro**	Low-CF-MiPro	Median-CF-MiPro	Raw	MiPro	Low-CF-MiPro	Median-CF-MiPro
**Akkermansiaceae**	1.29	0.543	0.00864	0.0113	0.399	10.7	0.716	0.0674
**Bacteroidaceae**	3.41	4.94	2.86	1.42	9.9	11.6	12.2	7.06
**Bifidobacteriaceae**	6.87	0.268	1.44	1.37	7.9	0.723	1.61	2.65
**Clostridiaceae**	4.61	54.6	43.5	54.6	3.82	9.6	12.8	11.1
**Enterobacteriaceae**	14.5	17.6	34.8	24.1	8.93	32.2	54.9	62.5
**Lachnospiraceae**	42.9	8.58	1.6	0.398	39.2	8.1	0.948	0.429
**Lactobacillaceae**	1.4	0.233	10.9	14.7	0.323	0.0923	0.311	2.73
**Pseudomonadaceae**	3.05	0.0432	0.0438	0.0201	0.317	0.492	2.18	0.258
**Ruminococcaceae**	1.57	0.236	0.0196	0.00318	7.77	0.277	0.0623	0.0212
**Veillonellaceae**	1.91	0.312	0.074	0.0142	0.738	1.41	2.07	4.38

*"Raw" represents the average relative abundance of the indicated phylum in uncultured colonoscopy aspirate or homogenized stool.

**Each medium condition represents the average relative abundance of the indicated phylum across all days of passage (1-5) in that medium.

Table S10. Statistical analyses of relative abundance of the top ten families as determined by mixed-effect linear modeling with Patient set as random effect.

Family	Genotype	Fixed effect	t-value	p-value	Significance code*
**Akkermansiaceae**	CF	Medium: low-CF	-1.61	0.11	NS
		Medium: med-CF	-1.6	0.11	NS
		Day	-2.5	0.013	*
	nonCF	Medium: low-CF	-6.42	8.45e-10	***
		Medium: med-CF	-6.84	8.12e-11	***
		Day	3	0.003	**
**Bacteroidaceae**	CF	Medium: low-CF	-2.44	0.016	*
		Medium: med-CF	-4.12	6.27e-05	***
		Day	1.04	0.30	NS
	nonCF	Medium: low-CF	0.54	0.59	NS
		Medium: med-CF	-3.91	0.00013	***
		Day	-0.53	0.60	NS
**Bifidobacteriaceae**	CF	Medium: low-CF	1.03	0.31	NS
		Medium: med-CF	0.97	0.33	NS
		Day	-3.98	0.00011	***
	nonCF	Medium: low-CF	0.92	0.36	NS
		Medium: med-CF	1.99	0.048	*
		Day	-5.92	1.23e-08	***
**Clostridiaceae**	CF	Medium: low-CF	-1.54	0.13	NS
		Medium: med-CF	0.004	0.99	NS
		Day	2.47	0.015	*
	nonCF	Medium: low-CF	1.29	0.2	NS
		Medium: med-CF	0.62	0.54	NS
		Day	2.73	0.0069	**
**Enterobacteriaceae**	CF	Medium: low-CF	3.27	0.0014	**
		Medium: med-CF	1.24	0.22	NS
		Day	2.18	0.031	*

	nonCF	Medium: low-CF	5.06	9.12e-07	***
		Medium: med-CF	6.75	1.35e-10	***
		Day	6.21	2.67e-09	***
**Lachnospiraceae**	CF	Medium: low-CF	-2	0.047	*
		Medium: med-CF	-2.34	0.02	*
		Day	-7.25	2.01e-11	***
	nonCF	Medium: low-CF	-2.76	0.0063	**
		Medium: med-CF	-2.96	0.0034	**
		Day	-9.4	<2e-16	***
**Lactobacillaceae**	CF	Medium: low-CF	2.72	0.0074	**
		Medium: med-CF	3.7	0.00031	***
		Day	3.07	0.0025	**
	nonCF	Medium: low-CF	0.27	0.79	NS
		Medium: med-CF	3.23	0.0014	**
		Day	-0.75	0.45	NS
**Pseudomonadaceae**	CF	Medium: low-CF	0.001	0.99	NS
		Medium: med-CF	-0.029	0.98	NS
		Day	-2.73	0.0072	**
	nonCF	Medium: low-CF	1.86	0.064	NS
		Medium: med-CF	-0.26	0.79	NS
		Day	-0.8	0.42	NS
**Ruminococcaceae**	CF	Medium: low-CF	-0.71	0.48	NS
		Medium: med-CF	-0.76	0.45	NS
		Day	-3.3	0.0012	**
	nonCF	Medium: low-CF	-0.34	0.73	NS
		Medium: med-CF	-0.41	0.69	NS
		Day	-8.71	7.67e-16	***
**Veillonellaceae**	CF	Medium: low-CF	-0.81	0.42	NS
		Medium: med-CF	-1.01	0.31	NS
		Day	-3.61	0.00041	***

	nonCF	Medium: low-CF	0.61	0.54	NS
		Medium: med-CF	2.76	0.0062	**
		Day	2.02	0.045	*

*Significant codes indicate: (NS: non-significant, *: p<0.05, **: p<0.01, ***: p<0.001)

Table S11. Relative abundance of top 15 genera across media conditions.

Genus	CF				nonCF			
	Raw*	MiPro**	Low-CF-MiPro	Median-CF-MiPro	Raw	MiPro	Low-CF-MiPro	Median-CF-MiPro
Akkermansia	1.27	0.543	0.00864	0.0113	0.399	10.7	0.716	0.0673
Bacteroides	3.41	4.94	2.86	1.42	9.9	11.6	12.2	7.06
Bifidobacterium	6.86	0.267	1.43	1.37	7.9	0.706	1.6	2.65
Blautia	18.4	1	0.162	0.31	13.9	1.47	0.233	0.199
Citrobacter	0	0.2	3.5	0.00353	0.011	0.534	0.579	0.0121
Clostridium sensu stricto 1	4.58	53.3	43.5	54.6	3.76	9.2	12.8	11.1
Enterococcus	0.181	3.83	2.36	1.48	0.58	3.58	3.91	2.88
Escherichia/Shigella	11.4	16.6	20.7	20.3	7.99	28.7	32.9	45.9
Faecalibacterium	0.611	0.189	0.00125	0.0016	5.95	0.00716	0.0146	0.0101
Klebsiella	2.89	0.405	8.93	3.36	0.711	1.41	18.9	15.1
Lactobacillus	0.456	0.0718	3.36	6.33	0.143	0.0205	0.0206	0.951
Pseudomonas	3.05	0.0429	0.0438	0.0201	0.317	0.492	2.17	0.258
Ruminococcus gnavus group	9.57	2.51	0.167	0.00857	5.28	0.627	0.0464	0.0605
Streptococcus	5.25	1.33	0.924	0.404	2	1.21	0.321	1.28
Veillonella	1.07	0.0147	0.0024	0.0142	0.562	0.461	1.65	4.36

*"Raw" represents the average relative abundance of the indicated phylum in uncultured colonoscopy aspirate or homogenized stool.

**Each medium condition represents the average relative abundance of the indicated phylum across all days of passage (1-5) in that medium.
