# Supplementary material for: An in vitro medium for modeling gut dysbiosis associated with cystic fibrosis
Source: J Bacteriol. 2024 Jan 3;206(1):e00286-23. doi: 10.1128/jb.00286-23 (PMC10810206; doi:10.1128/jb.00286-23)
Supplement: Supplemental figures — Fig. S1 to S9. [file jb.00286-23-s0002.pdf]

Supplemental Figures

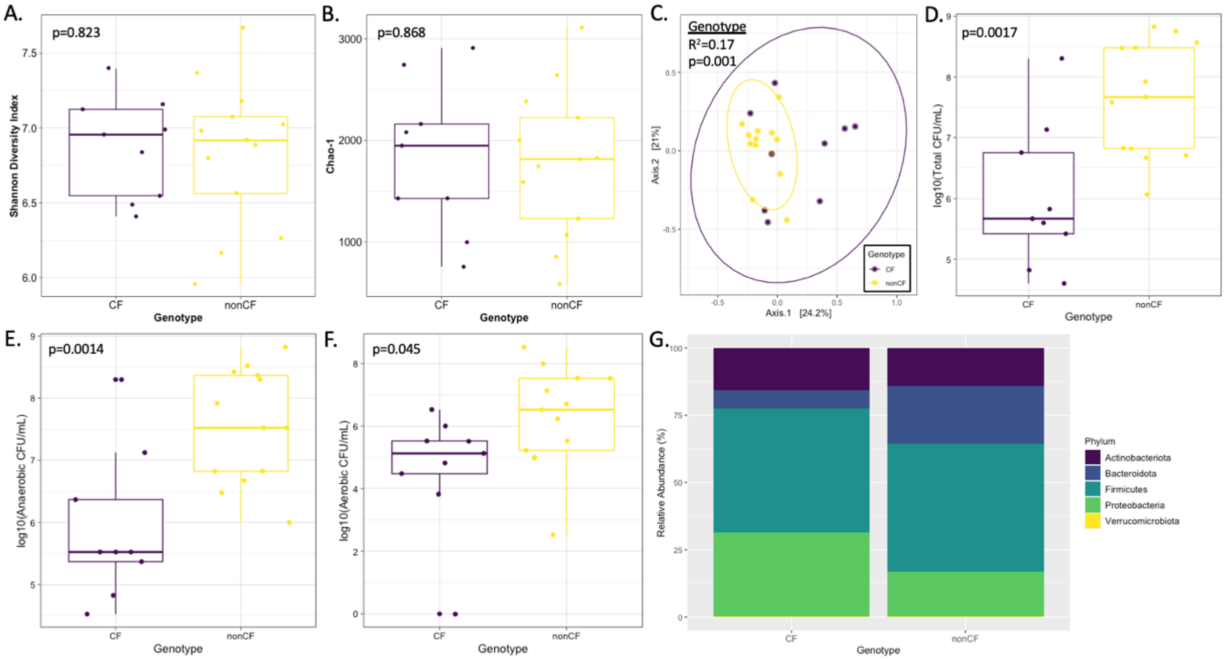

Supplemental Figure 1. Description of CF and nonCF stool or colonoscopy aspirates used as a source of the inoculum in the studies presented here. Alpha diversity of CF and nonCF stool and/or colonoscopy aspirates using (**A**) Shannon Diversity Index (SDI) or (**B**) Chao-1 distance. A linear model from the R package stats was used to test whether SDI or Chao-1 changed significantly with genotype. Neither SDI nor Chao-1 are significantly affected by genotype. (**C**) Bray-Curtis beta diversity was calculated for each sample and displayed on a principal coordinate analysis (PCA) plot, colored by genotype. The first two components account for 45.2% of total variance. Significant differences in beta diversity due to genotype were tested by PERMANOVA (p=0.001). (**D**) Sum CFU/mL were calculated following growth on blood sheep agar at (**E**) 0% or (**F**) 21% oxygen. A linear model from the R package stats was used to test whether CFU/mL from each oxygen tension changed significantly with genotype. CF samples culture less CFU/mL at 0% (**E**) and 21% oxygen (**F**), thus less total CFU/mL compared to nonCF samples (**D**). **(G)** Relative abundance of the top five phyla, legend to the right, of CF and nonCF samples. In CF samples, Proteobacteria (green) is increased and Bacteroidota (blue) is decreased.

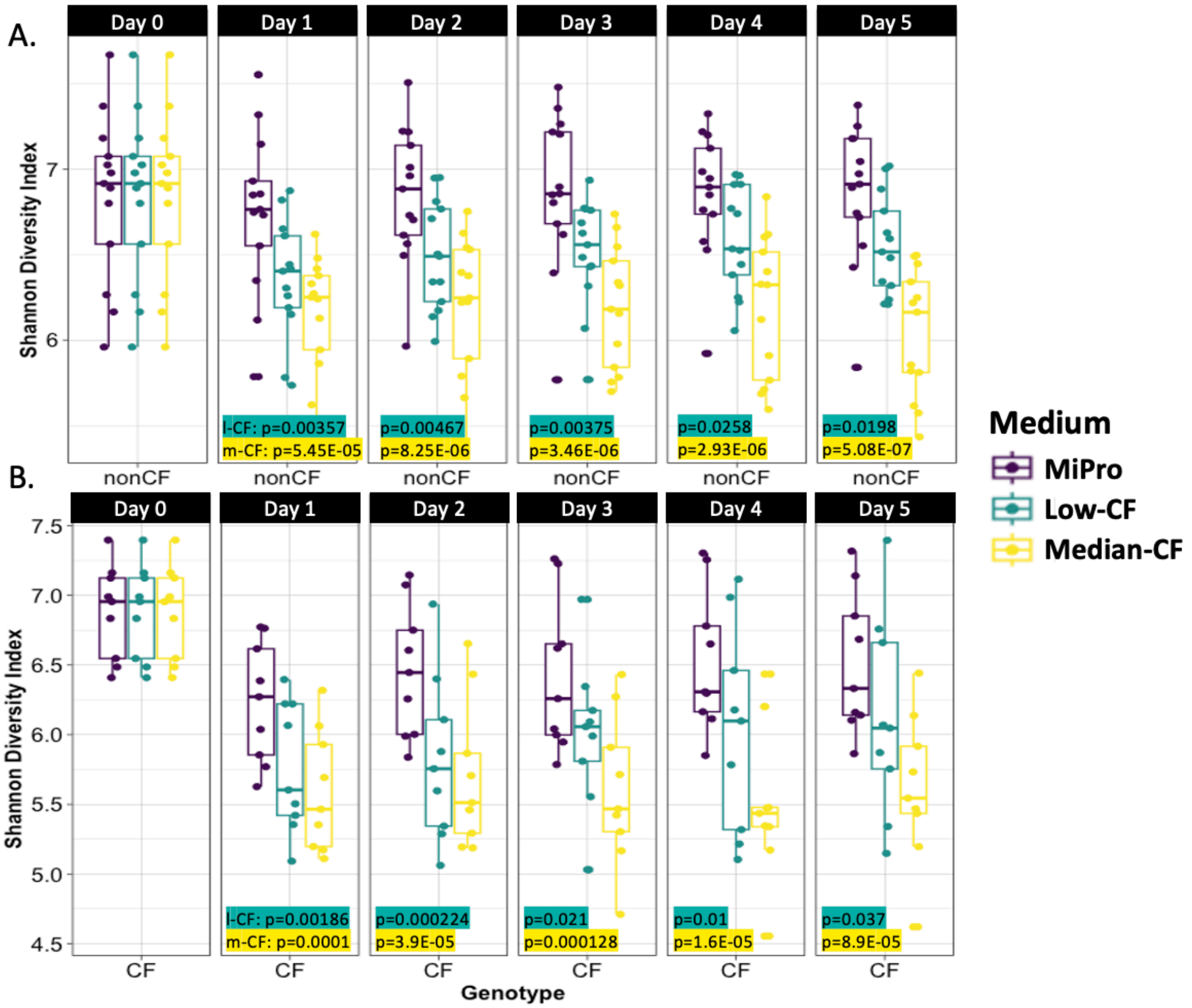

Supplemental Figure 2. Shannon Diversity Index (SDI) across each day of passage. SDI for (A) nonCF and (B) CF samples passaged in each medium for five days. A linear mixed effect model was used to test whether SDI changed significantly with medium type within each day of passage. Patient was set as the random variable to control for multiple sampling. SDI is significantly reduced in CF-MiPro, in a dose-dependent manner, at each day of passage in both genotypes, suggesting the feasibility of shorter passage durations.

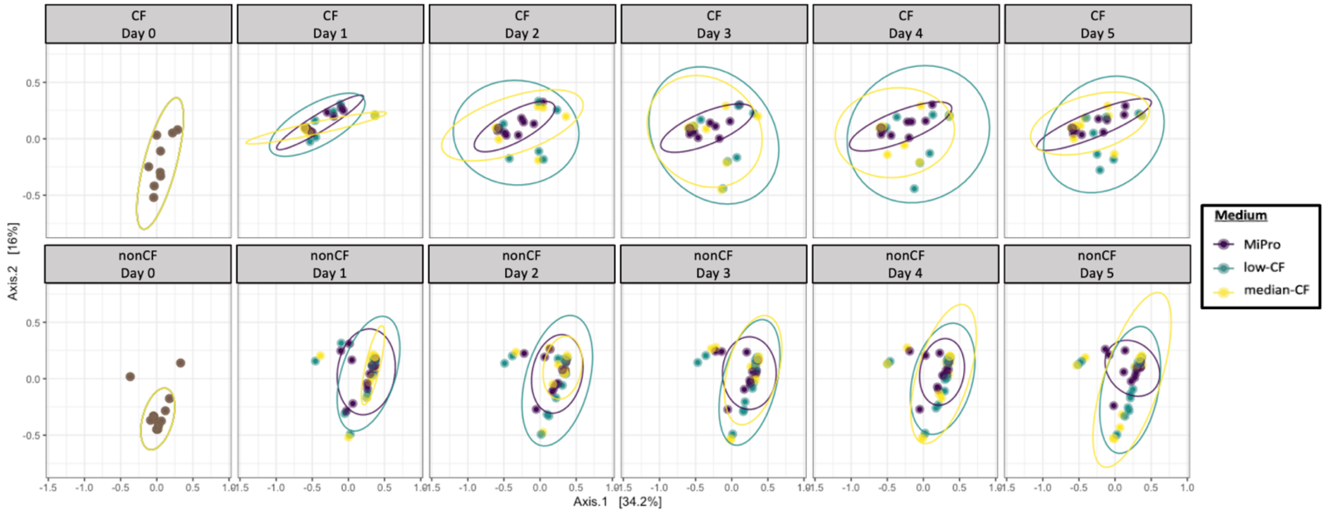

Supplemental Figure 3. Bray-Curtis beta diversity of CF and nonCF samples at each day of passage. Bray-Curtis beta diversity was calculated for each (top) CF and (bottom) nonCF sample and displayed on PCoA plots, faceted by genotype and day of passage and colored by medium. Shifts in microbial composition occur largely at Day 1 for both genotypes, with additional shifts attributed to culture condition ("Medium").

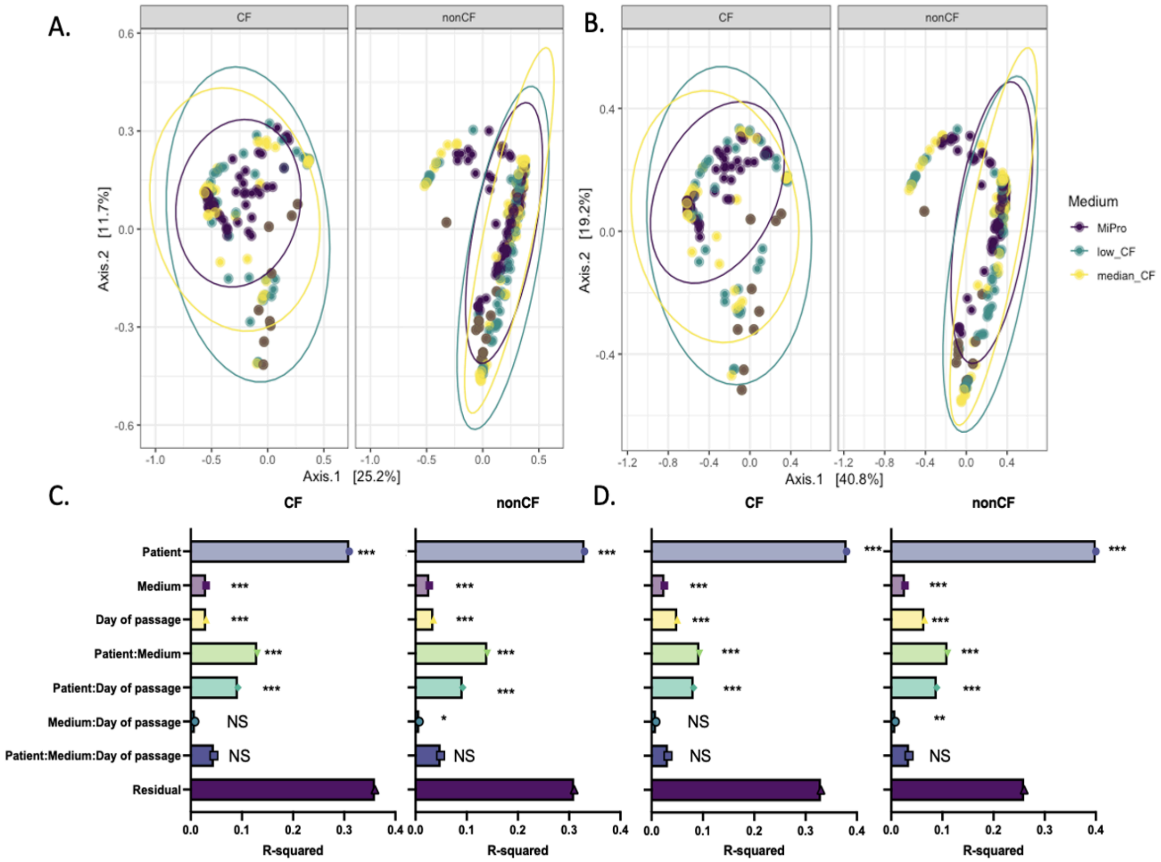

Supplemental Figure 4. Jaccard and Morisita-Horn beta diversity of CF and nonCF samples cultured through MiPro and CF-MiPro. (**A**) Jaccard and (**B**) Morisita-Horn beta diversity was calculated for each sample and displayed on PCA plots, faceted by genotype and colored by medium. The first two components account for (A) 36.9% and (B) 60% of total variance. Statistical differences in (**C**) Jaccard and (**D**) Morisita-Horn beta diversity were tested by PERMANOVA with metadata included in the model. R-squared values for all data included, as well as potential interactions, are plotted with significance codes indicated (NS: non-significant, *: p<0.05, **: p<0.01, ***: p<0.001). “Residual” indicates variation unexplained by the model.

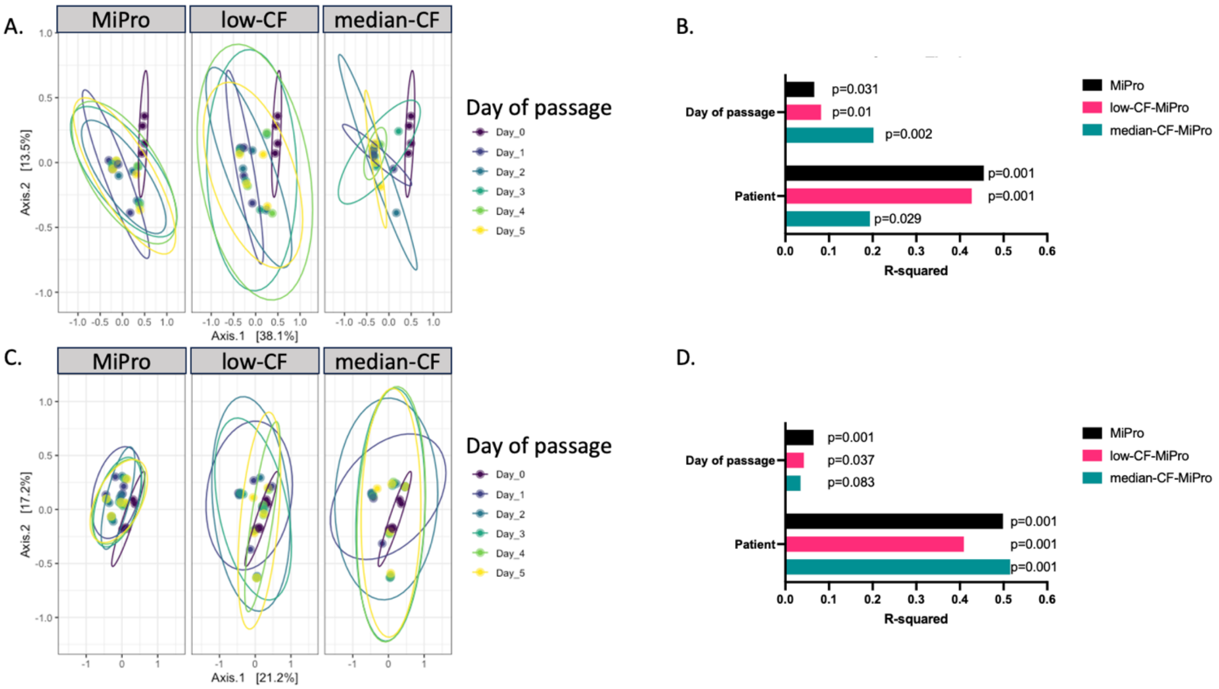

Supplemental Figure 5. Bray-Curtis beta diversity of CF Day 0-5 samples homogenized in L-cysteine or glycerol. Bray-Curtis beta diversity was calculated for each sample prepared in PBS supplemented with (**A-B**) 10 mM L-cysteine or (**C-D**) 7.15% glycerol and displayed on PCoA plots, faceted by medium and colored by Day of passage. (B and D) Statistical differences in Bray-Curtis beta diversity were tested by PERMANOVA, with metadata included in the model. R-squared values for all data included are plotted with p-values indicated.

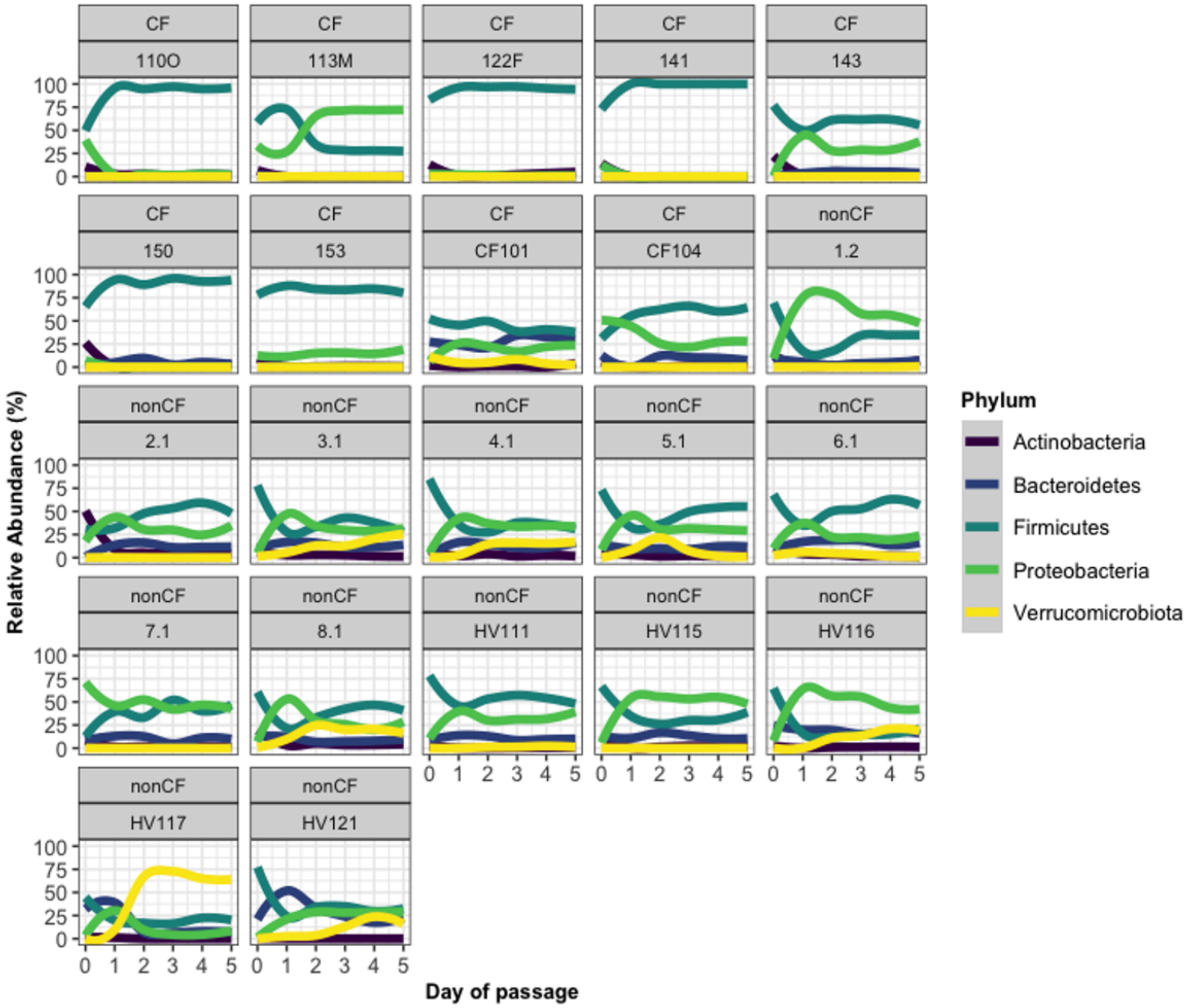

Supplemental Figure 6. Intra-person variation of microbial relative abundance changes in MiPro. Day of passage is graphed versus relative abundance of the indicated taxa for each sample, and a linear plot was used to visualize overall changes in microbial relative abundance at the phylum level during in vitro passages in MiPro. The legend indicates the taxonomic assignment for each panel. Taxonomical shifts occur largely at Day 1 and are not restricted to a specific genotype, sample type (stool or colonoscopy aspirate), homogenization protocol (“Prep”: glycerol or cysteine) or sequencing batch.

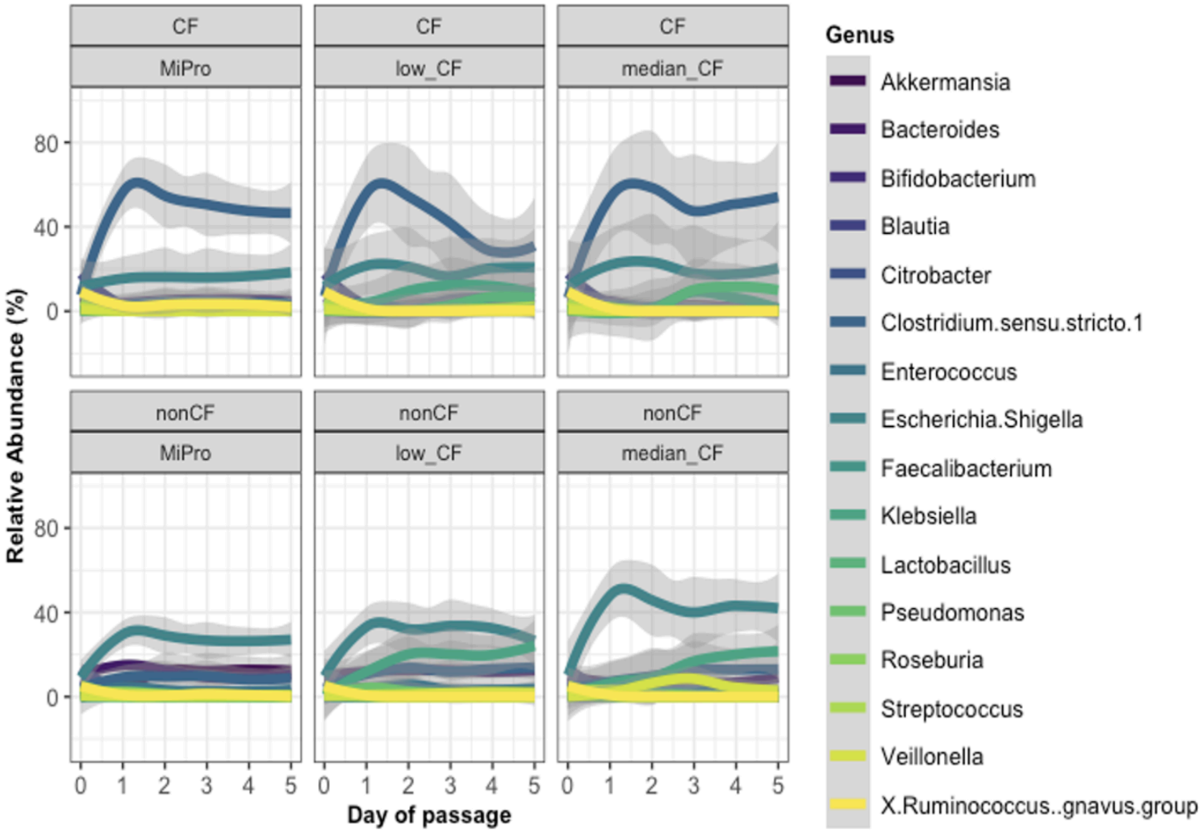

Supplemental Figure 7. Genus relative abundance across all days of passage. Day of passage is graphed versus relative abundance of the indicated taxa for each sample, and a linear plot was used to visualize overall changes in microbial relative abundance at the genus level across all media conditions. Samples originating from a CF donor are displayed in the top panels, and samples originating from a nonCF donor are displayed in the bottom panels. The legend indicates the taxonomic assignment for each panel. Note: many of the genera are low in abundance and the lines overlap and cannot be discerned in this figure. Only the genera that show robust changes are easily seen.

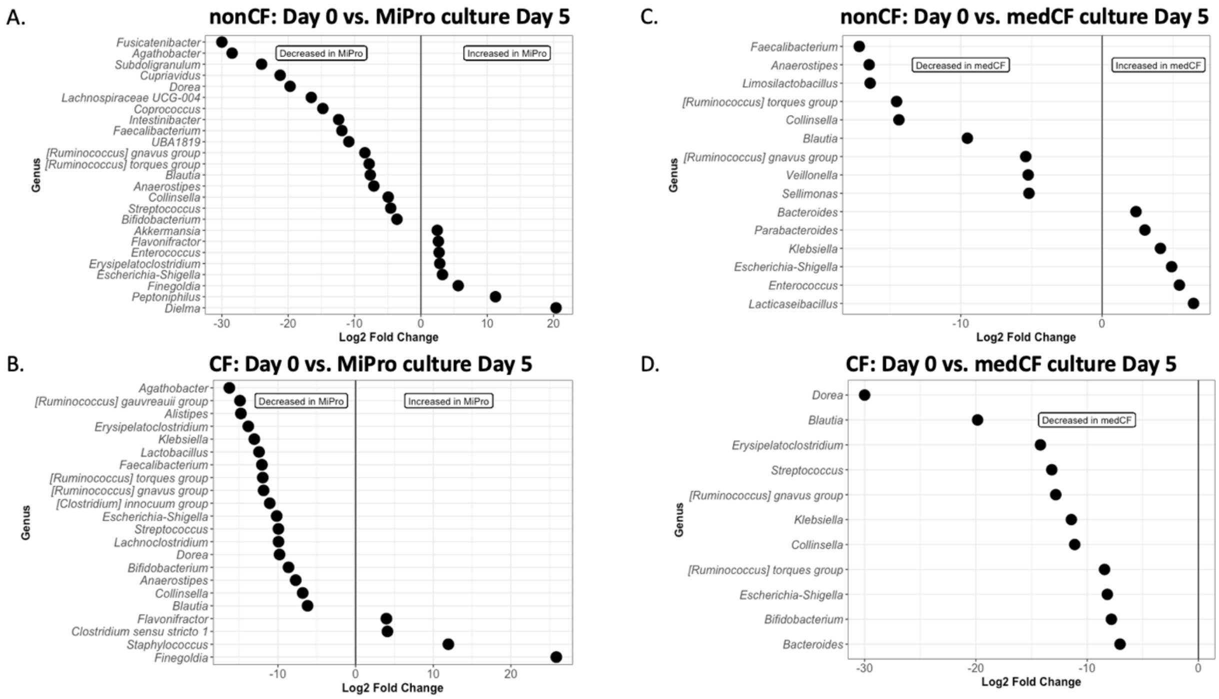

Supplemental Figure 8. Log_2 fold change of taxa that were significantly altered in cultured samples versus raw samples. Log_2 fold change of taxa that were significantly (p-adj.<0.05) altered in (**A**) nonCF and (**B**) CF samples passaged in MiPro, Day 5 versus uncultured, Day 0, or (**C**) nonCF and (**D**) CF samples passaged in median-CF-MiPro, Day 5 versus uncultured, Day 0. Taxa were filtered to be present in at least 5% of all samples. Each dot represents a single genus. Significance was determined by DESeq2 using a non-continuous model of samples binned by day of passage (0 vs. 5) within each media condition. Patient was included as a design variable to control for multiple sampling.

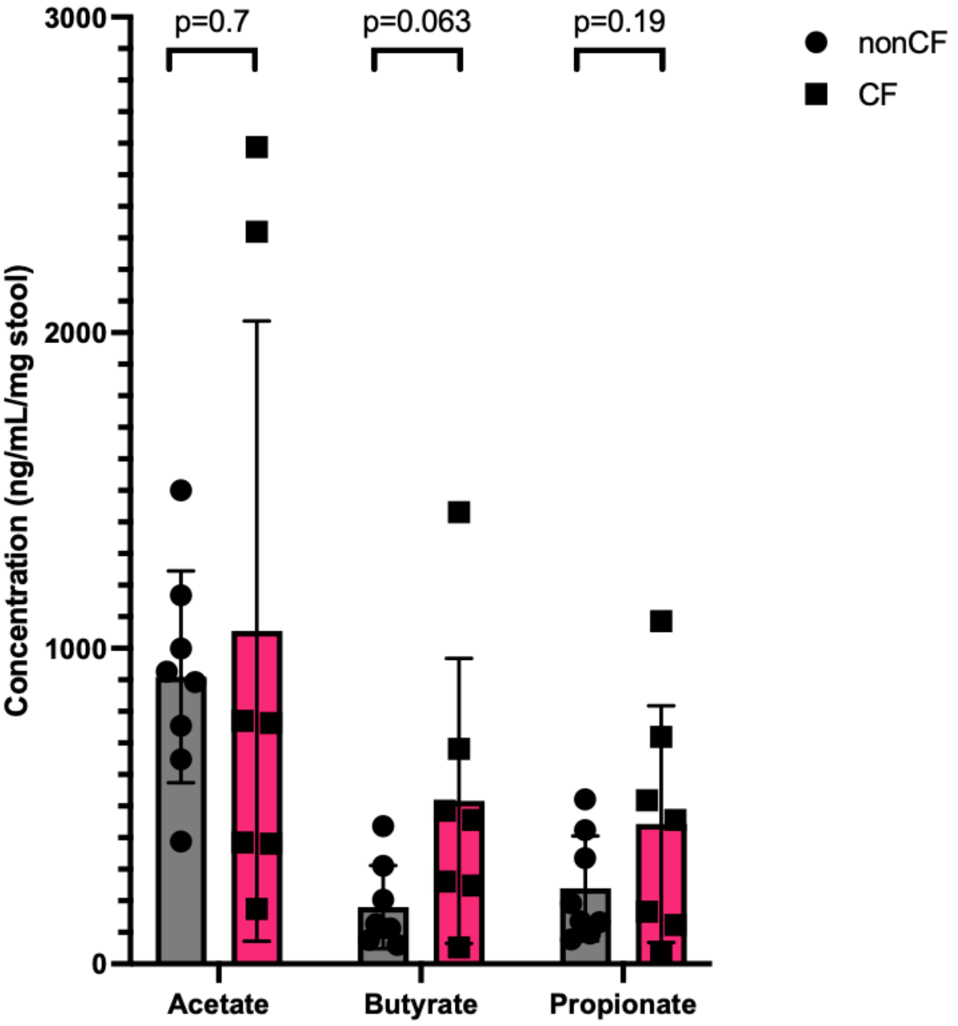

Supplemental Figure 9. Baseline short chain fatty acid (SCFA) concentrations in CF and nonCF stool samples. Major SCFA, including acetate, butyrate and propionate, were quantified using GC/MS from raw stool samples (nonCF: n=8, CF: n=7). Metabolite concentrations were normalized by stool weight (mg) and volume of extraction buffer (mL of methanol). Linear regression was used to determine statistical significance of genotype on SCFA concentrations.
