# Supplementary material for: An in vitro medium for modeling gut dysbiosis associated with cystic fibrosis
Source: J Bacteriol. 2024 Jan 3;206(1):e00286-23. doi: 10.1128/jb.00286-23 (PMC10810206; doi:10.1128/jb.00286-23)
Supplement: Supplemental methods — Protocol: in vitro passaging with CF-MiPro. [file jb.00286-23-s0003.pdf]

Protocol: In vitro passaging with CF-MiPro

I. At least one day prior to start of experiment:

1. Make MiPro, low- and median-CF-MiPro with all components, excluding glycerol, nitrate, sulfate, formate, hydrogen peroxide and antibiotic. Autoclave on liquid, 45 minute cycle. Store at rt.
2. Let media cool to room temperature, then de-gas in an anaerobic chamber* for at least 24h prior to start of experiment.
3. Place 5 (or as many as needed) sterile 12-well plates in anaerobic chamber to de-gas for at least 24h prior to start of experiment.

*Anaerobic chamber conditions: 37°C Whitley A55 anaerobic chamber (Don Whitley Scientific, Victoria 412 Works, UK) with gas composition of 10% CO_2, 10% H_2, 80% N_2

II. Day of experiment:

4. Thaw gut microbiome sample (stool or colonoscopy aspirate) on ice.
5. Pre-weigh an empty 15 mL Falcon tube.
6. Nitrogen-purge homogenization solution (sterile PBS supplemented with 10 mM L-cysteine or 7.15% glycerol) for 10-15 min.
7. Once sample is thawed, aliquot into a tared conical tube, weigh.
    a. Note: stool samples must be homogenized, however colonoscopy aspirates are relatively dilute and do not require further dilution.
8. Homogenize stool sample in a ratio of 1:5.7 w/v (example: 1 g stool = 5.7 mL PBS + cysteine or glycerol) in de-gassed homogenization solution. Vortex, then spin at 715 x *g* in a table-top centrifuge for 15 seconds to drop large chunks.
    a. This sample will be used to inoculate into each medium and is referred to as "Day 0".
9. In the anaerobic chamber, add remaining components to low- and median-CF-MiPro (glycerol, nitrate, sulfate, formate, hydrogen peroxide and antibiotic), mix well, then aliquot 3.92 mL of medium into a well of the sterile Costar 12-well plate.
    a. Include three extra wells of medium only for contamination controls.
10. Using Day 0 samples, mix with pipette and add 80 uL to each well (to bring the final volume to 4 mL).
11. On Day 0, follow Part III for processing and storage of sample.
12. On Day 1 (24h following start of experiment), repeat step 9.
13. For the first 12-well plate set up on Day 0, mix each well by pumping 50x using a P1000 pipette.
14. Sub-culture 80 uL of the mixed sample into fresh medium. Collect remaining sample in a 15 mL conical for processing (Part III).
15. For each consecutive day, repeat steps 9-14.

III. Prep of Day 0-5 samples:

16. In a 96-well plate, serially dilute contamination controls and Day X samples (10-fold), and plate for CFU/mL on 2 sheep blood agar plates. Incubate plates at 37°C (21% oxygen and 0% oxygen) for 24h.
17. With the remaining Day X sample, spin at 5,000 x *g* for 3 min.
18. Pour supernatant into a clean 15 mL conical. Spin at 5,000 x *g* for 5 min at 4°C. Filter sterilize supernatant using 0.22 μM filter and syringe. Store at -80°C until further metabolite analysis.
19. Following QIAGEN's protocol, add 2 mL of RNAprotect Bacteria Reagent to cell pellet, vortex. Let sit at room temperature for 5 min.
20. Spin mixture at 5,000 *x g* for 10 min. Aspirate supernatant. Store at -80°C until DNA extraction.

IV. DNA extraction using Zymo Quick-DNA Fecal/Soil Microbe MiniPrep Kit

Table 1. Medium recipe**.**

Component**	Stock solution		Final concentration		Volume stock solution/Liter	
CF-MiPro Formulation	*Low*	*Median*	*Low*	*Median*	*Low*	*Median*
Stomach porcine mucin	N/A		6 g/L	8 g/L	6 g	8 g
Bile acid mix	N/A		1 g/L	2 g/L	1 g	2 g
MES (buffer)	195.24 g/mol		100 mM	100 mM	19.524 g	19.524 g

Adjust pH to 6 using HCl and/or NaOH, autoclave, cool then add:

Component**	Stock solution		Final concentration		Volume stock solution/Liter	
CF-MiPro Formulation	*Low*	*Median*	*Low*	*Median*	*Low*	*Median*
Glycerol	10%	20%	0.5%	1%	50 mL	50 mL
Sodium nitrate	20 mM	40 mM	0.5 mM	1 mM	25 mL	25 mL
Sodium sulfate	20 mM	40 mM	0.5 mM	1 mM	25 mL	25 mL
Sodium formate	20 mM	40 mM	0.5 mM	1 mM	25 mL	25 mL
Hydrogen peroxide	1 mM	10 mM	1 uM	10 uM	1 mL	1 mL
Bactrim (antibiotic)	1 mM	10 mM	1 uM	10 uM	1 mL	1 mL

**Any of these components can be omitted or altered as desired. All components should be added fresh on the day of experimentation.
